# Predicting Subnational Ebola Virus Disease Epidemic Dynamics from Sociodemographic Indicators

**DOI:** 10.1371/journal.pone.0163544

**Published:** 2016-10-12

**Authors:** Linda Valeri, Oscar Patterson-Lomba, Yared Gurmu, Akweley Ablorh, Jennifer Bobb, F. William Townes, Guy Harling

**Affiliations:** 1 Psychiatric Biostatistics Laboratory, McLean Hospital, Belmont, United States of America; 2 Harvard Medical School, Boston, United States of America; 3 Department of Biostatistics, Harvard T.H. Chan School of Public Health, Boston, United States of America; 4 Department of Epidemiology, Harvard T.H. Chan School of Public Health, Boston, United States of America; 5 Group Health Research Institute, Seattle, United States of America; 6 Department of Global Health and Population, Harvard T.H. Chan School of Public Health, Boston, United States of America; Curtin University, AUSTRALIA

## Abstract

**Background:**

The recent Ebola virus disease (EVD) outbreak in West Africa has spread wider than any previous human EVD epidemic. While individual-level risk factors that contribute to the spread of EVD have been studied, the population-level attributes of subnational regions associated with outbreak severity have not yet been considered.

**Methods:**

To investigate the area-level predictors of EVD dynamics, we integrated time series data on cumulative reported cases of EVD from the World Health Organization and covariate data from the Demographic and Health Surveys. We first estimated the early growth rates of epidemics in each second-level administrative district (ADM2) in Guinea, Sierra Leone and Liberia using exponential, logistic and polynomial growth models. We then evaluated how these growth rates, as well as epidemic size within ADM2s, were ecologically associated with several demographic and socio-economic characteristics of the ADM2, using bivariate correlations and multivariable regression models.

**Results:**

The polynomial growth model appeared to best fit the ADM2 epidemic curves, displaying the lowest residual standard error. Each outcome was associated with various regional characteristics in bivariate models, however in stepwise multivariable models only mean education levels were consistently associated with a worse local epidemic.

**Discussion:**

By combining two common methods—estimation of epidemic parameters using mathematical models, and estimation of associations using ecological regression models—we identified some factors predicting rapid and severe EVD epidemics in West African subnational regions. While care should be taken interpreting such results as anything more than correlational, we suggest that our approach of using data sources that were publicly available in advance of the epidemic or in real-time provides an analytic framework that may assist countries in understanding the dynamics of future outbreaks as they occur.

## Introduction

The recent outbreak of Ebola virus disease (EVD) in humans in West Africa is believed to have begun in the Guinean prefecture of Guéckédou, located in the southern Nzérékoré region in late 2013 [1, 2]. It subsequently spread to geographically contiguous Lofa County in Liberia and Kailahun District of Sierra Leone in early 2014 [3]. Over the following year, the EVD epidemic reached almost every part of these three countries [4], as well as briefly transmitting in Nigeria, Senegal, Mali and the United States [5–8].

The nature of this spread has been described geographically [9, 10], anthropologically [11, 12] and genetically [2, 13]. In terms of socioeconomic and demographic factors, several authors have highlighted the link between EVD’s spread and the low level of resources available to the healthcare system in these three countries [14–16]. A recent analysis mapped mean multidimensional, household-level poverty across Liberian districts, and highlighted an apparent positive correlation between the Social Vulnerability Classification they produced and EVD outbreak severity [17]. It has also been noted that take-up of behaviour change messages was central to epidemic control [18]. Furthermore, mathematical models have also been used to examine the transmission dynamics and spreading potential of this outbreak at the subnational [19, 20], national [21–24] and international levels [10]. However, a systematic investigation of how demographic and socio-economic factors might predict differential EVD dynamics at the subnational level has yet to be conducted.

The trail of human and economic devastation left by this Ebola outbreak has left policymakers with many concerns about how to effectively cope in the future with a rapidly evolving epidemic such as the West African EVD outbreak. Notably, planning response efforts requires accurate assessments of: (i) how likely an outbreak is to occur in each part of a country; (ii) how rapidly it is likely to grow; and (iii) how large the overall outbreak is likely to be. This epidemic represents the first time that such a large, geographically and culturally heterogeneous area has been affected at one time by this disease. It therefore also presents the first opportunity to explore how EVD epidemic dynamics differ by area-level characteristics that are readily available to planning authorities.

Area-level characteristics that might be expected to predict epidemic dynamics include: (1) demographics, given age [25] and sex [26] differences in EVD incidence and mortality; (2) education, given the importance of behavior change message uptake; (3) population density and urbanicity, given the interpersonal transmission route of EVD; (4) religious practices and burial rites [27]; and (5) wealth, as a proxy at the community level for both economic activity that might increase risk, and resources available to protect against infection spread.

We conducted an analysis of the initial growth rates and cumulative incidence of the EVD epidemic in all second-order administrative units (ADM2) within the three most-affected countries (regions of Guinea; Counties of Liberia; Districts of Sierra Leone). We aimed to identify which demographic and socio-economic characteristics were associated with rapid growth or large overall epidemic size, and then link these findings to the existing literature on the epidemic to help determine which areas within countries might be at greatest risk of being affected by EVD in a subsequent outbreak.

## Materials and Methods

In the present study we propose a two stage approach. In the first stage we estimate area-level epidemic growth rates, and then in a second stage we investigate which area-level factors are associated with the epidemic growth and final number of affected cases. We conducted our analyses at the second-order administrative unit level (ADM2) in each country. This level includes the eight regions of Guinea, the 14 districts of Sierra Leone and the 15 counties of Liberia. We excluded ADM2 units which had reported no cases of EVD up to 31 July 2015 (Labe, Guinea; Maryland, Liberia). Thus we used a total of 35 ADM2 units.

### Data sources

We employed time series data on EVD cumulative reported cases at the subnational level in Guinea, Sierra Leona, and Liberia, obtained from the World Health Organization [28]. In our analysis, the final epidemic size was the cumulative number of cases in each of the subnational regions as of the end of July 2015. Since the epidemic continued in some regions after this date, these quantities are an approximation to the true *final* epidemic size.

We obtained area-level covariate data from the most recent Demographic and Health Surveys (DHS) in each of the three countries (Guinea: 2012; Liberia: 2013; Sierra Leone: 2013). DHS studies are surveys conducted through a stratified two-stage sampling process; weights provided by the DHS allow the calculation of representative figures within each region of a country, based on the age-strata eligible for interview (15-49 year old women; 15-59 year old men, except in Liberia where men were also aged 15-49). Details on each study are available through final survey reports [29–31]. We used DHS variables for gender (% female), religion (% Christian, Muslim or Other) urbanicity (% living in an urban sampling unit), and mean age and mean number of years of education as potential predictors of the epidemic dynamic. All variables were aggregated to generate weighted means or percentages at the ADM2 level based on sampling and response rates as provided by DHS reports, and further adjusted for gender-specific sampling based on United Nations population estimates for 2015 [32]. Values therefore represent ADM2 characteristics amongst core working-aged adults.

In addition, we included economic wellbeing as measured by the mean International Wealth Index (IWI) value for each ADM2 [33, 34], and population size and density based on the most recently available national Census data on each country’s National Bureau of Statistics website [35–37]. Finally, we included a variable for how soon the first EVD case in each ADM2 was reported, relative to the start of the West African outbreak.

### Epidemic growth modeling

We estimated early growth rates to characterize the initial rise of the EVD epidemic in each ADM2 in Guinea, Sierra Leone and Liberia. Since WHO case count updates occurred irregularly in time, we linearly interpolated between any two consecutive updates separated by more than seven days, to obtain estimates for weekly cumulative incidence.

We fit growth models to the resulting time series of weekly cumulative case counts in each ADM2. To investigate the sensitivity of the growth rate estimates to modeling assumptions, we considered three curve fitting models featured in the recent literature on modeling EVD epidemic growth rates: exponential growth [24], polynomial growth [19] and logistic growth [21]. In addition, to test the sensitivity of the growth curve parameter estimates to the number of data points used for fitting the curves, we used two time windows: one from the first observed case in an ADM2 up to 6 weeks after (6 data points); the other up to 15 weeks (15 data points). Next, we describe in detail the three growth models, and highlight their strengths and limitations in modeling the epidemic growth.

#### Exponential Growth

A common and parsimonious assumption is that the cumulative number of cases grows exponentially during the early stages of the epidemic, particularly when the susceptible population is very large and no interventions have been put in place [24, 38]. The statistical model proposed here to describe the early growth of cumulative cases is:

$$\begin{array}{c} C_{i}\left(t\right)=c_{e}\exp\left(r_{e}t\right)+\epsilon_{i}, \end{array}$$ (1)

where *C*_*i*_(*t*) is the cumulative number of cases in region *i* by week *t*, and *ϵ*_*i*_ is an error term. Via curve fitting methods (nonlinear least squares method nls2, **R** software [39]), we can obtain point estimates for *c*_*e*_ and *r*_*e*_, and their respective standard errors. Since our main goal is to quantify the early epidemic rise, we are most interested in estimates of *r*_*e*_.

#### Polynomial Growth

**Fig 1:**
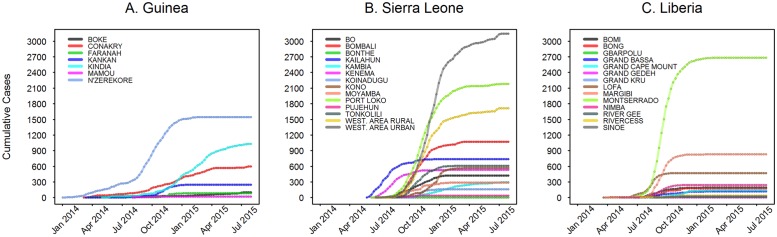
Weekly cumulative time series data at the ADM2 subnational level. Plots for time since first case in each ADM2. A. Guinea; B. Sierra Leone; C. Liberia.

It was recently noted that, even though the national cumulative curves of EVD cases in Guinea, Sierra Leone and Liberia followed an approximate exponential growth curve, the early growth patterns of EVD outbreaks at the subnational-level were better approximated by a polynomial function [19], which could be driven by high clustering in the underlying contact network or host behavioral changes [20]. Hence, we also used a polynomial growth model to fit the data:

$$\begin{array}{c} C_{i}\left(t\right)=c_{1}+c_{2}t^{m}+\epsilon_{i}, \end{array}$$ (2)

where parameters *c*_1_, *c*_2_ and *m* can be estimated through least-squares regression. It is clear that the higher the exponent *m* is, the higher the growth rate of the cumulative cases. However, the polynomial growth assumption has the added advantage that the *rate* of increase can either increase (convex behavior) or decrease (concave behavior) with time, whereas the exponential assumption with a fixed rate *r*_*e*_ can only represent a convex behavior. To show this briefly, note that *C* ∼ *t*^*m*^, thus *dC*/*dt* ∼ *mt*^*m*−1^. Hence, when *m* > 1, the rate of change increases with time (convex) and when *m* < 1, the rate of change decreases with time (concave). This flexibility is suitable for modeling EVD in West Africa since the early growth phase in some regions is better described by concave functions, and in others by convex functions (Fig 1).

#### Logistic Growth

The logistic growth model is often used in population biology to describe a population that grows under limited resources [40]. Pathogens are also, in a sense, a population of “predators” feeding off a finite population of susceptible individuals that decreases over time (due to depletion). Thus, the logistic model can provide, in principle, a suitable description of the outbreak trajectory. A recent work used a logistic growth model to describe and quantify the current EVD outbreak [21]. The statistical model in this case is:

$$\begin{array}{c} C_{i}\left(t\right)=\frac{1}{1/k+c_{l}\exp\left(-r_{l}t\right)}+\epsilon_{i}, \end{array}$$ (3)

where *k* is the carrying capacity in the population biology context, and in this epidemiological context can be interpreted as the final epidemic size (the cumulative number of infected cases at the end of the epidemic); *c*_*l*_ is a shift parameter; and *r*_*l*_ quantifies the intrinsic growth rate of the epidemic. Via the same curve fitting method mentioned above we can obtain estimates of these three parameters, although we are particularly interested in *r*_*l*_.

For these three models, we measure goodness of fit using the residual standard error:

$$\begin{array}{c} RSE=\sqrt{\frac{RSS}{n-p-1}} \end{array}$$

where *RSS* is the residuals sums squared, and *n* − *p* − 1 are the degrees of freedom of a model with *n* data points and *p* parameters (thus penalizing model complexity). Models with smaller *RSE* fit the data more appropriately.

### Statistical analyses of area-level factors associated with epidemic growth and size

We investigated bivariate correlations among the socioeconomic predictors and five outcomes: the three growth rate estimates from the three aforementioned models, final epidemic size and final epidemic proportion (epidemic size divided by population size). To evaluate potential predictors of these outcomes we first investigated the magnitude and significance of pairwise correlations for each time window (6 and 15 weeks post-arrival of EVD in an ADM2) using Spearman rank correlations.

We next employed a step-wise backward variable selection procedure based on the Bayesian Information Criterion (BIC) [41] to fit parsimonious multivariable linear regression models, using the command step() in R [42]. Reported coefficient estimates and p-values are from each final selected model. Our models were of the form:

$$\begin{array}{c} Y_{i}=\beta_{0}+\sum_{p=1}^{10}\beta_{p}X_{pi}+\epsilon_{i} \end{array}$$ (4)

with *i* = 1,2…,35 ADM2 regions, where the outcome variable *Y*_*i*_ was either the polynomial, exponential or logistic growth rate, respectively *PolyGrowth*_*i*_, *ExpGrowth*_*i*_ and *LogGrowth*_*i*_, or the final epidemic size *EpidSize*_*i*_, or the final epidemic proportion *EpidProp*_*i*_. *X*_*p*_ with *p* = 1,..,10 denoted the set of selected predictors. As a sensitivity analysis, we further adjusted for country in the final models.

## Results

### Curve fits and growth rates

In Fig 1 we show the complete time series of the epidemic at the ADM2 level in Guinea, Sierra Leone, and Liberia up to the end of July 2015. As observed previously [19], the outbreaks within each country occurred asynchronously, with outbreak onset likely related to the geographic and transportation network structure of the regions. In addition, the rates of increase and final epidemic size show great variability between regions.

Goodness of fit analyses suggest that the polynomial model performed the best out of the three modeling functions used to fit the early time series data, in agreement with previous observations [19] (S4 Fig). We therefore focused on the growth rates obtained from the polynomial fit in our subsequent analyses; results for the exponential and logistic models are shown in S2 Fig.

**Fig 2:**
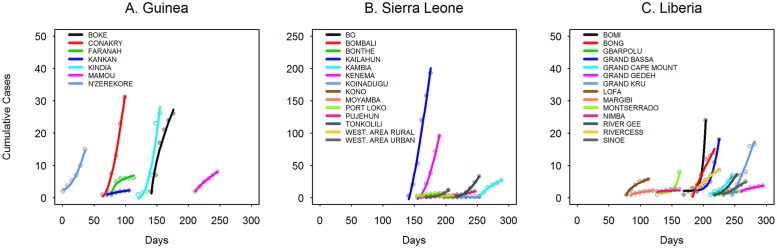
Polynomial model fit to the first 6 weeks of cumulative time series data at the subnational level for each country. Time zero is the date of the first reported EVD case for the whole outbreak in Guinea. A. Guinea; B. Sierra Leone; C. Liberia.

Fig 2 shows the polynomial model fit for the early epidemic in each ADM2 (6 weeks corresponds to approximately 3 disease generations, given a mean generation time of 15 days [19, 43]). To analyze the sensitivity of these parameter estimates, we additionally estimated them using up to 15 weeks of data from regional onset (see S1–S5 Figs).

### Descriptive Statistics and Correlation analysis

**Fig 3:**
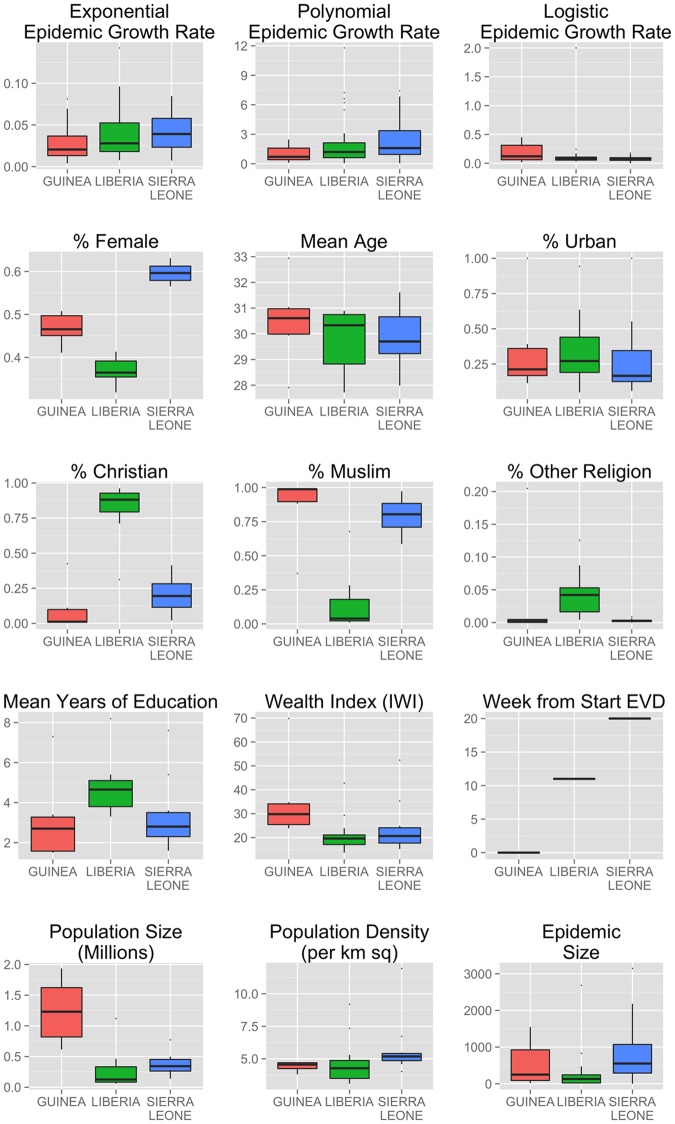
Boxplots of dependent and independent variables. Each plot shows mean, interquartile range and any outlier values; all are measured at the ADM2 subnational level.

We first inspected the distribution of outcomes and covariates across the three countries (Fig 3 and S5 Fig). Estimated rates were heterogeneous, with lower epidemic growth rates in Guinea in the exponential and polynomial models, but a higher rate in Guinea based on the logistic model. Large heterogeneities were seen for gender and religious composition, as well as in level of education and wealth across countries. The Liberian population has a higher percentage of Christian population, is more educated on average, and has a lower female gender ratio. Guinea is more populous and wealthier on average. Sierra Leone is the most densely populated country, with Freetown the most densely populated ADM2.

**Fig 4:**
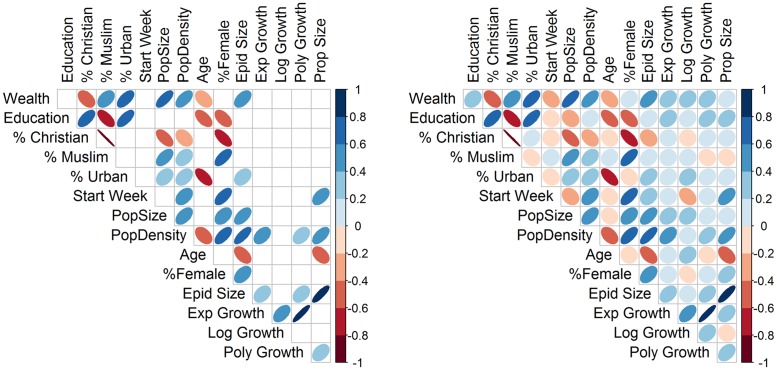
Pairwise Spearman correlation plots between outcomes and covariates. The direction of correlation is indicated by color type (blue: positive; red: negative). The strength of correlation is indicated by color intensity; uncertainty around the estimates is indicated by width of the ovals (wider: more uncertainty). A. Only correlations statistically significant at *α* = 0.05. B. All correlations. Outcomes: regional growth rate estimated using data from first 15 weeks of epidemic in each region (exponential fit, “*ExpGowth*”; logistic fit, “*LogGrowth*”; polynomial fit, “*PolyGrowth*”); total number of infections reported in each region (“*EpidSize*”), proportion of whole population infected (“*EpidProp*”). Socio-economic covariates: wealth index, “*Wealth*”; average years of education, “*Education*”; percent of population Christian, “%*Christian*”; percent of population Muslim, “%*Muslim*”; percent of population living in urban area, “%*Urban*”; number of weeks from start of EVD in West Africa to the first recorded case in each region, “*StartWeek*”; population size, “*PopSize*”; population density, “*PopDensity*”; average age, “*Age*”; percent of population female, “%*Female*”).

Correlation analyses (Fig 4, S1 Table and S6 Fig) show strong positive correlations between growth rates estimated from exponential and polynomial models, using either 6 weeks or 15 weeks of time series data. Final epidemic size was strongly correlated with the growth rates from exponential and polynomial curves based on 15 weeks of data, but not when using only 6 weeks. Growth rates estimated from the logistic model were not associated with any of the other outcomes or ecologic predictor variables, and had poor model fit. In subsequent regression analyses we focus mainly on interpreting results regarding the rates obtained from the polynomial model, as these appear more reliable.

Urbanicity, wealth and education were positively correlated. Age was found negatively correlated with wealth, education, and urbanicity. Education was positively correlated with percentage Christian. Overall, covariates were more strongly correlated with final epidemic size and epidemic proportion than with growth rates.

### Multivariable regression model

The step-wise procedure for the final epidemic size and epidemic proportion models selected two predictors that were positively correlated with both outcomes: percentage female and mean education level (see Table 1). Of note, the adjusted R-squared of the model for epidemic size was higher than for the models predicting growth rates (*Adj* *R*^2^ = 0.50 for final epidemic size versus *Adj* *R*^2^ = 0.24 for polynomial growth rates).

**Table 1:** Multivariable linear regression model for growth rates from exponential, logistic and polynomial models (6 weeks of data), final epidemic size and final epidemic proportion, adjusting for all predictors selected by step-wise backwards regression using the BIC criterion.

	*Dependent variable:*				
	exponential	logistic	polynomial	epidemic size	epidemic percentage
	(1)	(2)	(3)	(4)	(5)
Wealth index				−43.4**	
				(−78.1, −8.74)	
Mean education (years)	0.01*		0.99***	449***	0.06***
	(−0.000, 0.01)		(0.32, 1.67)	(258, 639)	(0.03, 0.10)
Percent urban			−0.04*		
			(−0.08, 0.005)		
Population (thousands)			0.001	1.00***	
			(−0.000, 0.002)	(0.41, 1.58)	
Population density (per km^2^)				0.01**	
				(0.001, 0.02)	
Percent female			0.04	45.5***	0.01***
			(−0.02, 0.09)	(25.7, 65.3)	(0.004, 0.01)
Constant	0.03**	0.17***	−3.25	−2,726***	−0.53***
	(0.003, 0.05)	(0.06, 0.28)	(−7.08, 0.58)	(−3,929, −1,523)	(−0.88, −0.19)
Observations	35	35	35	35	35
Adjusted R^2^	0.077	0.000	0.214	0.640	0.293
Residual Std. Error	0.027	0.331	1.343	471.118	0.161
F Statistic	3.823*		3.315**	13.080***	8.047***

Note: Values are point estimates and 95% confidence intervals.

*p<0.1;

**p<0.05;

***p<0.01.

F statistic is from a test that all coefficient values are jointly equal to zero. Outcome units: equations (1-2) 1/time; (3) unitless; (4) count number of reported cases; (5) percentage points of population reported as cases. Covariate units: education, one additional year; percent female, ADM2 is one additional percentage point female.

Step-wise procedure using BIC criterion for exponential and logistic growth rates did not select any covariate, while Education was selected as predictor of faster polynomial growth rates. The same factor was selected in the epidemic size and epidemic proportion models. “Percent female” was also found to be associated with larger epidemic size and epidemic proportion. Model fit for the polynomial model was notably better than for the other two growth models. The variable selection procedure did not select country fixed effects. However, as a sensitivity analysis we adjusted for country fixed effects in all models (S2 Table). In these latter models, the only change was to the effect of “Percent female” in the models for epidemic size and epidemic proportion, which had an increased effect size but a much increased variance, leaving the variable non-significant in the model.

## Discussion

In an effort to improve our understanding of how socioeconomic factors explain heterogeneity in disease dynamics across areal units, we have combined two common analytic approaches: (1) estimation of epidemic parameters using mathematical models; and (2) estimation of associations between epidemic outcomes and potential predictors using multiple regression at the ecological (not individual) level. Our goals in this paper were: first, to present a novel way of matching together curve-fitting models and empirical data; and second, to suggest possible correlates of underlying risk factors, in case these commonly measured sociodemographic features may correlate similarly with underlying factors in the future. In the context of the 2013-15 Ebola virus disease outbreak in West Africa, we show that some of these measures were indeed predictive of higher cumulative disease incidence and of faster disease outbreak.

**Fig 5:**
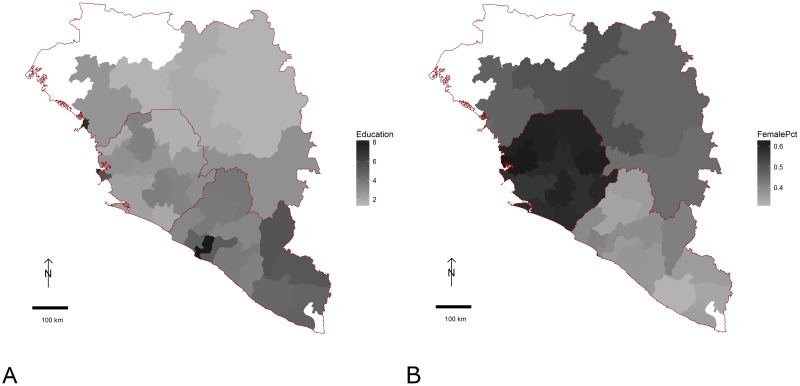
Geographic distribution of covariates across ADM2 units in Guinea, Liberia and Sierra Leone. A: Mean education in years. B: Proportion of 18-49 year old population female. White regions are those which reported no EVD cases in the study period.

Notably, we find that more educated areas of Guinea, Liberia and Sierra Leone had more severe EVD outbreaks as measured by speed of epidemic growth and final epidemic size. This was true in both bivariate and multivariable analysis. To make sense of this finding, we note that three other factors strongly associated with education—wealth and urbanicity and thus population density—were positively associated with the epidemic in bivariate analysis, but subsequently appeared negatively associated once education was accounted for. This cluster of covariates all highlight that the fastest epidemic take-off and largest final sizes were seen in and around the three capital cities, i.e., Conakry, Monrovia and Freetown, which also have the highest average education levels (Fig 5A). All of these variables may thus be acting as proxies for causal effects arising from the closer proximity of households and individuals in urban areas, or differences in how people reacted to the epidemic or control efforts enacted to fight the epidemic. These factors may also reflect the later arrival of the epidemic in these urban settings, compared to the more-rural settings from which the outbreak emerged.

Our initial finding that a higher female:male population ratio was associated with a larger epidemic was instructive in the care needed using automated procedures. Although our stepwise selection model did not indicate the need to include country-level indicator variables in predicting epidemic outcomes, visual inspection of the sex ratio values (Fig 5B) made it clear that there were large country-level variations in estimated sex ratios. Once we included country-level indictors (S2 Table), the putative association remained, although less significantly than before. Interpreting this association is difficult: while there was not a clear sex-difference in infection rates in this outbreak [44], the association we show is at the group level, and thus any causal interpretation would also need to be at this level.

Our association analysis has parallels with the more data-intensive, real-time, digital early-warning systems proposed by others [45]. While these digital analyses are likely to provide dynamic predictions of risk during an epidemic, our simpler, statical analysis may be more widely applicable—even in the absence of rapidly updated predictor information.

As well as highlighting potential predictors of EVD epidemic severity, this study highlights once again the sensitivity of curve-fitting exercises to the assumptions made in the model specification process. We would encourage others to test alternative methods, to determine which risk factors are robustly associated with epidemic severity. We would also encourage those with other datasets or area-level variables that they expect to predict EVD risk to test them in similar models. Given the importance of ecological niches for disease vectors [46], remote sensor data might prove a fruitful avenue for investigation, as might communications patterns seen in cellphone metadata [47] and road transport flow volumes. One possible extension of our regression analysis might be to explicitly model the spatial dependence between contiguous ADM2s seen in disease spread during the epidemic [48].

### Strengths and limitations

As has been noted by others, the data available during this outbreak have been of varying quality [49] and often not openly available [50]. First, there was almost certainly under-ascertainment of cases during this EVD outbreak due to resource constraints and the similarity of EVD symptoms to other prevalent infections in the area, including malaria and non-EVD hemorrhagic fevers. Second, there have been significant and variable time lags between when cases were first found on the ground, and when they were added to national and then World Health Organization databases [51]. Model estimates should not be strongly affected so long as this mis-reporting was random across space and time, however systematic differences in reporting rates may lead to misleading estimates of epidemic growth rates. For example, mis-reporting might vary systematically between urban and rural areas. One might expect lower rural reporting rates if lower access to medical facilities or transport led to non-reporting of rural cases; alternatively, weaker traditional leadership encouragement of case reporting, or greater distrust of government, might lead to lower urban reporting rates. Any conclusions drawn about associations seen in this paper should be tempered by such uncertainty.

The nature of the available data may require different modeling assumptions. Arguably, one of the strengths of this work is that by modeling the area-level epidemic growth with three different models we were able to assess how sensitive are the growth rate estimates to the different assumptions implicit in the models, which is critical for the usefulness and applicability of the methodology and results presented herein [21]. For example, the polynomial fit outperformed the exponential and logistic models, and correlated more strongly with the final epidemic sizes, because in some areas where epidemics had in general a relative small number of cases, a convex growth model (such as the one intrinsic to the exponential and logistic models) was not able to capture the concave trajectory of the cumulative case data. These observations certainly support the use of the polynomial growth model to estimate the rates of area-level epidemics spread, however, a limitation of this approach is that the polynomial model is less interpretable than the exponential or logistic models in terms of factors characterizing the underlying dynamics [19].

An important caveat to the curve-fitting exercise in this analysis is that cumulative incidence data can be highly inter-correlated from point to point, and thus violate the independence assumption of standard least squares methods. As a result, the estimates obtained from fitting to cumulative incidence data can potentially lead to biased results [24]. It is also important to note that in some cases the least square algorithm had difficulties converging to parameters estimates for the curve-fitting exercise. This difficulty varied by region, growth model and data range. In such cases, the cumulative case curves followed growth patterns that did not resemble the respective growth models. However, even for these “problematic” cases we were still able to find sensible parameter estimates by employing a parameter space exploration method available in nls2. Recently, Viboud and colleagues introduced a generalized-growth model to characterize the early rise of infectious outbreaks, which is designed to capture epidemic growth profiles ranging from sub-exponential to exponential [52]. This alternative epidemic growth estimation method can be readily adopted within the framework we propose in this paper.

In interpreting our findings, it is important to bear in mind that this analysis was intentionally exploratory in nature. There are many other pre-existing data sources that could extend our analysis, and we would highlight that any associations we found should not be interpreted causally. It is also crucial to interpret our findings as ecological in nature, since we did not have individual-level data. Thus the associations shown hold at the ADM2 level, rather than suggesting anything about individual level risk factors, which have been analyzed elsewhere [53]. There are also clearly many other potential ecological predictors of EVD severity that could have been included in the model, and thus our results are open to potential confounding by other factors. Furthermore, the DHS data used for calculating age and sex values at the ADM2 level sample only from adults of reproductive age (15-59 years) and therefore may not fully capture the demographic profile of each region.

Epidemic dynamics observed during this outbreak did not occur in a vacuum; efforts were made to prevent infections, and to isolate and treat contagious individuals. Observed epidemic values were thus not independent of interventions, which may have differed depending on resource availability and timing of outbreak (e.g., areas affected later on saw more rapid responses). Thus the associations shown with sociodemographics variables should be interpreted not as reflecting the “natural” dynamics of EVD, but rather its variability in the context of an evolving response. However, it may be reasonable to assume that variability in epidemic outcomes across areas reflects (at least to some extent) characteristics of these areas. And insofar as variable response efforts reflected variation in political will or in ability to access areas, these variations are of substantive interest.

### Conclusions

We have laid out a framework for analyzing epidemic dynamics in a setting where, and at a time when, data were at a premium. We combine existing, publicly available data sources with real-time epidemic information to generate hypotheses as to which areas might be at greatest risk for EVD epidemic take-off and overall impact. Our approach can be conducted rapidly during an epidemic outbreak—particularly one of a novel disease context such as that seen in 2013-15 in West Africa—to provide policymakers with information that can be used to target key interventions across large-scale geographic areas. While this information cannot provide the individual-level detail that clinical records [54] or viral genotypes [2] can, it has the potential to provide information at a complementary, societal scale that may be valuable for public health, rather than medical, practice.

The methodology presented here can be refined and applied in other settings—in West Africa and beyond—which are at risk of EVD, to help countries anticipate where outbreaks might be most harmful, and allocate resources accordingly. We thus hope that the ideas presented in this paper are able to act as a spur to further efforts to understand the dynamics of the 2013-15 EVD epidemic and other epidemics as well to prevent the spread of future outbreaks.

## Supporting Information

S1 Fig Polynomial fit of weekly cumulative infection count using data from the first 15 weeks of the outbreak in each region. (PDF)

S2 Fig Exponential and logistic fit of weekly cumulative infection count using data from the first 15 weeks of the outbreak in each region. (PDF)

S3 Fig Comparison of log growth rate estimates from different models. (PDF)

S4 Fig Comparison of model Goodness of Fit from different models. (PDF)

S5 Fig Comparison of uncertainty of growth rate estimates from different models based on standard deviations. (PDF)

S6 Fig Correlation plots of dependent and independent variables. (PDF)

S1 Table Descriptive statistics for dependent and independent variables. (PDF)

S2 Table Multivariable linear regression models including country-level fixed effects. (PDF)

S1 Text Stata and R code required to replicate results shown in this paper. (DOCX)

S1 File CSV datasets of all data aside from Demographic and Health Study datasets. (ZIP)
