# Supplementary material for: Predicting Subnational Ebola Virus Disease Epidemic Dynamics from Sociodemographic Indicators
Source: PLoS One. 2016 Oct 12;11(10):e0163544. doi: 10.1371/journal.pone.0163544 (PMC5061396; doi:10.1371/journal.pone.0163544)
Supplement: S1 Fig — (PDF) [file pone.0163544.s001.pdf]

Predicting subnational Ebola virus disease epidemic dynamics from sociodemographic indicators

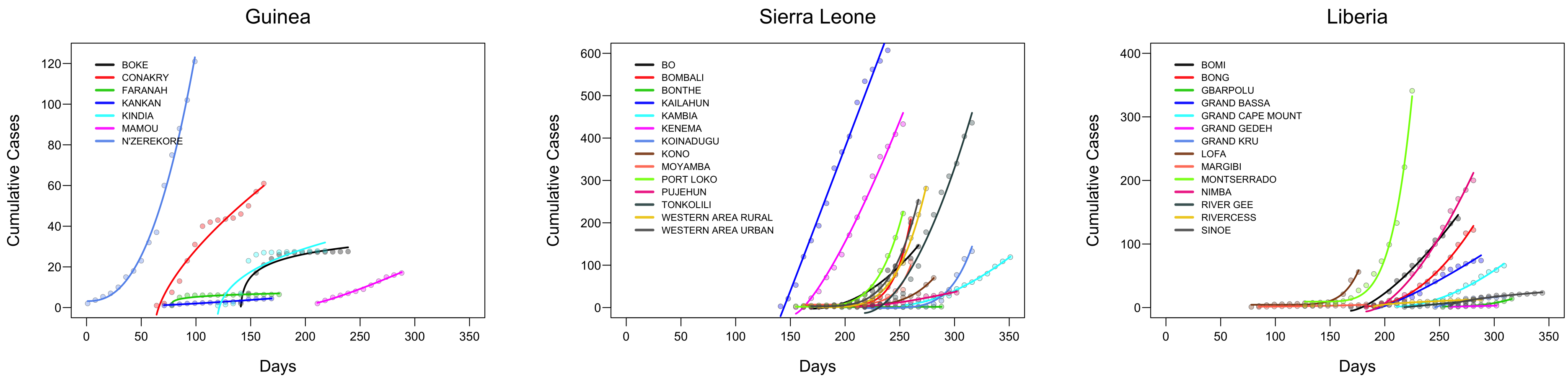

Figure S1. Polynomial fit to the weekly cumulative time series (model in equation 2) for all three countries at the subnational level. Using data from the first 15 weeks of the outbreak in each region.
