# Supplementary material for: Predicting Subnational Ebola Virus Disease Epidemic Dynamics from Sociodemographic Indicators
Source: PLoS One. 2016 Oct 12;11(10):e0163544. doi: 10.1371/journal.pone.0163544 (PMC5061396; doi:10.1371/journal.pone.0163544)
Supplement: S2 Fig — (PDF) [file pone.0163544.s002.pdf]

Predicting subnational Ebola virus disease epidemic dynamics from sociodemographic indicators

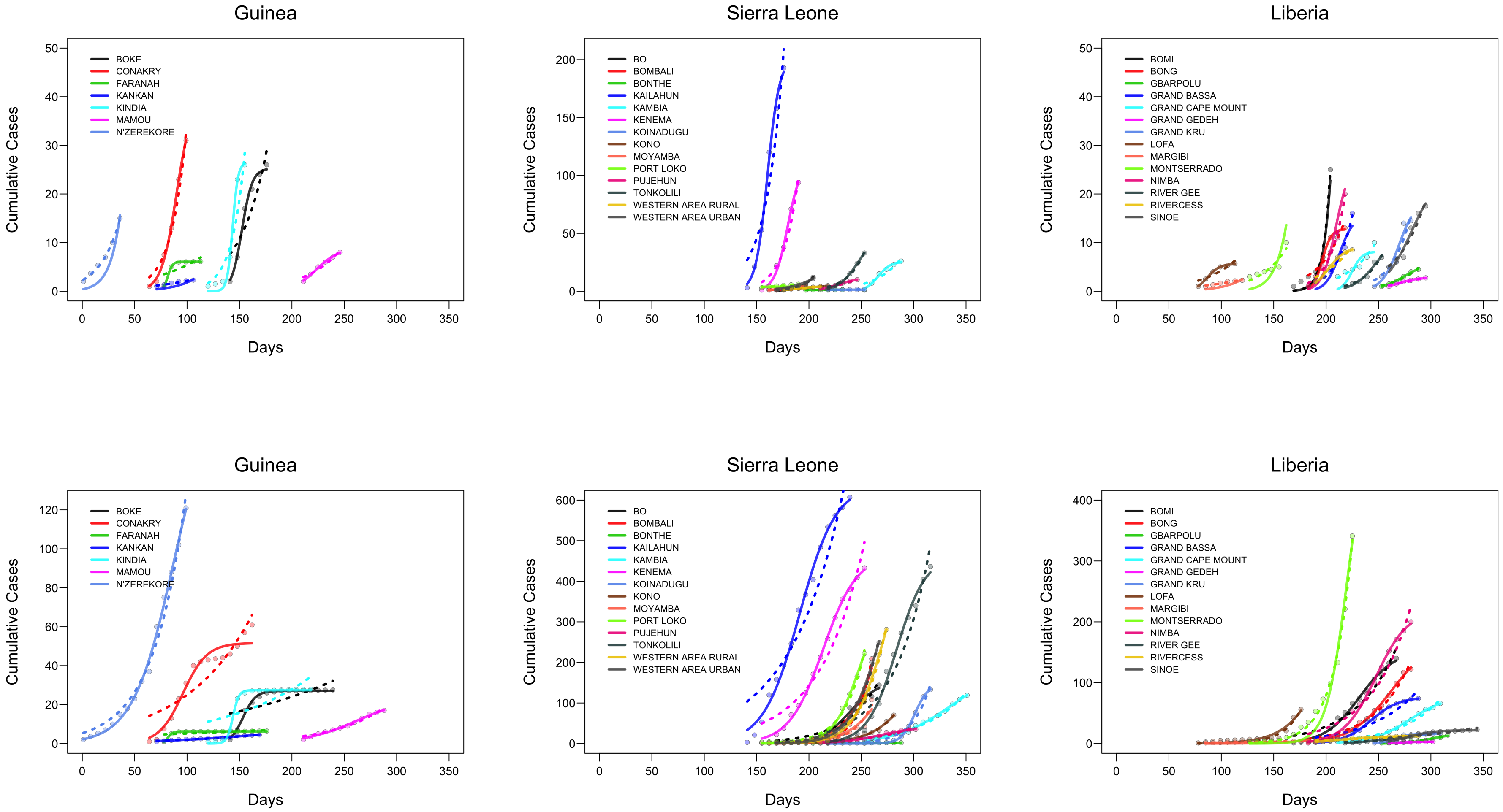

Figure S2. Exponential (dashed lines) and logistic (solid lines) fits to the weekly cumulative time series (model in equations 1 and 3 respectively) for all three countries at the subnational level. Using data from the first 6 weeks (upper panel) and 15 weeks (lower panel) of the outbreak in each region.
