# Supplementary material for: Predicting Subnational Ebola Virus Disease Epidemic Dynamics from Sociodemographic Indicators
Source: PLoS One. 2016 Oct 12;11(10):e0163544. doi: 10.1371/journal.pone.0163544 (PMC5061396; doi:10.1371/journal.pone.0163544)
Supplement: S3 Fig — (PDF) [file pone.0163544.s003.pdf]

Predicting subnational Ebola virus disease epidemic dynamics from sociodemographic indicators

Figure S3 displays the logarithm of the growth rates as estimated using the three different models, when using up to 6 weeks (upper panel) and up to 15 weeks of data (lower panel).

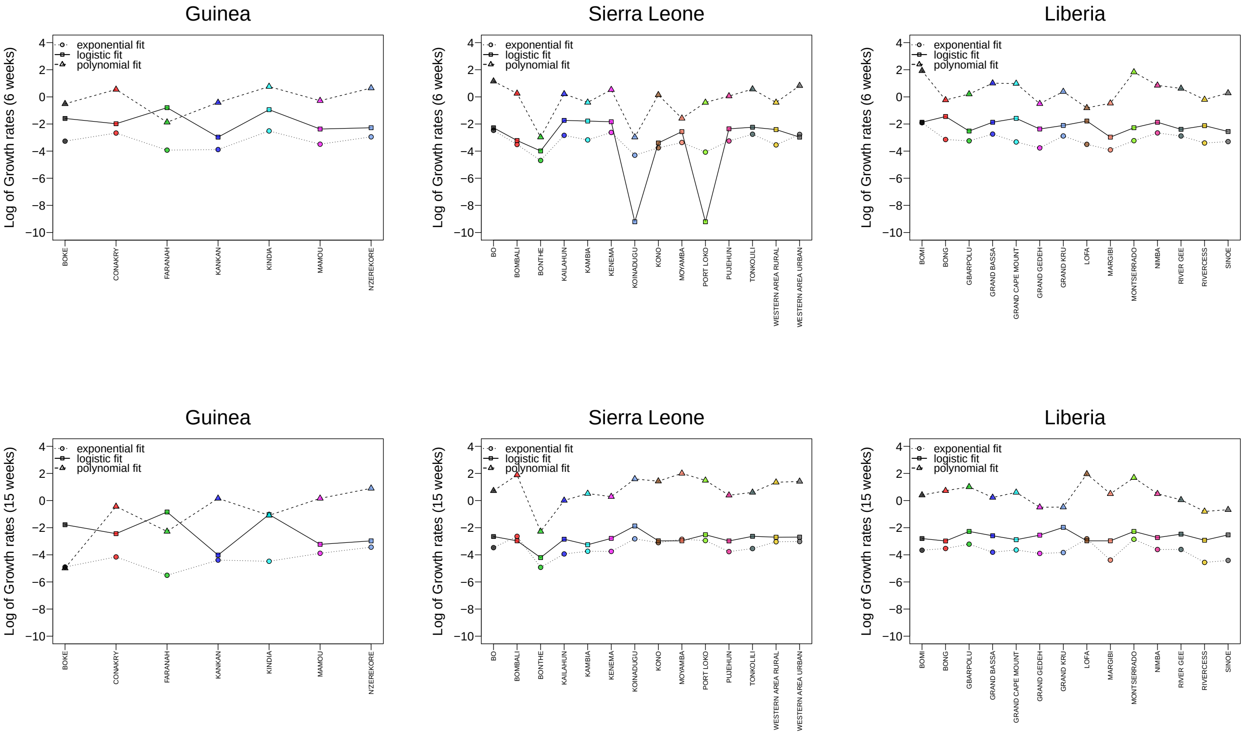

Figure S3. Comparing (log) growth rate estimates in each region, for each country. Using data from the first 6 weeks (upper panel) and 15 weeks (lower panel) of the outbreak in each region.
