# Supplementary material for: Predicting Subnational Ebola Virus Disease Epidemic Dynamics from Sociodemographic Indicators
Source: PLoS One. 2016 Oct 12;11(10):e0163544. doi: 10.1371/journal.pone.0163544 (PMC5061396; doi:10.1371/journal.pone.0163544)
Supplement: S4 Fig — (PDF) [file pone.0163544.s004.pdf]

Predicting subnational Ebola virus disease epidemic dynamics from sociodemographic indicators

We use residual standard error (RSE) to compare how well did the different models fit the data in different regions. Figure S4 displays the logarithm of the growth rates as estimated using the three different models, when using up to 6 weeks (upper panel) and up to 15 weeks of data (lower panel). On average, the best fit was obtained using the polynomial fit. As expected, increasing the number of data points from 6 to 15 increased the residual errors.

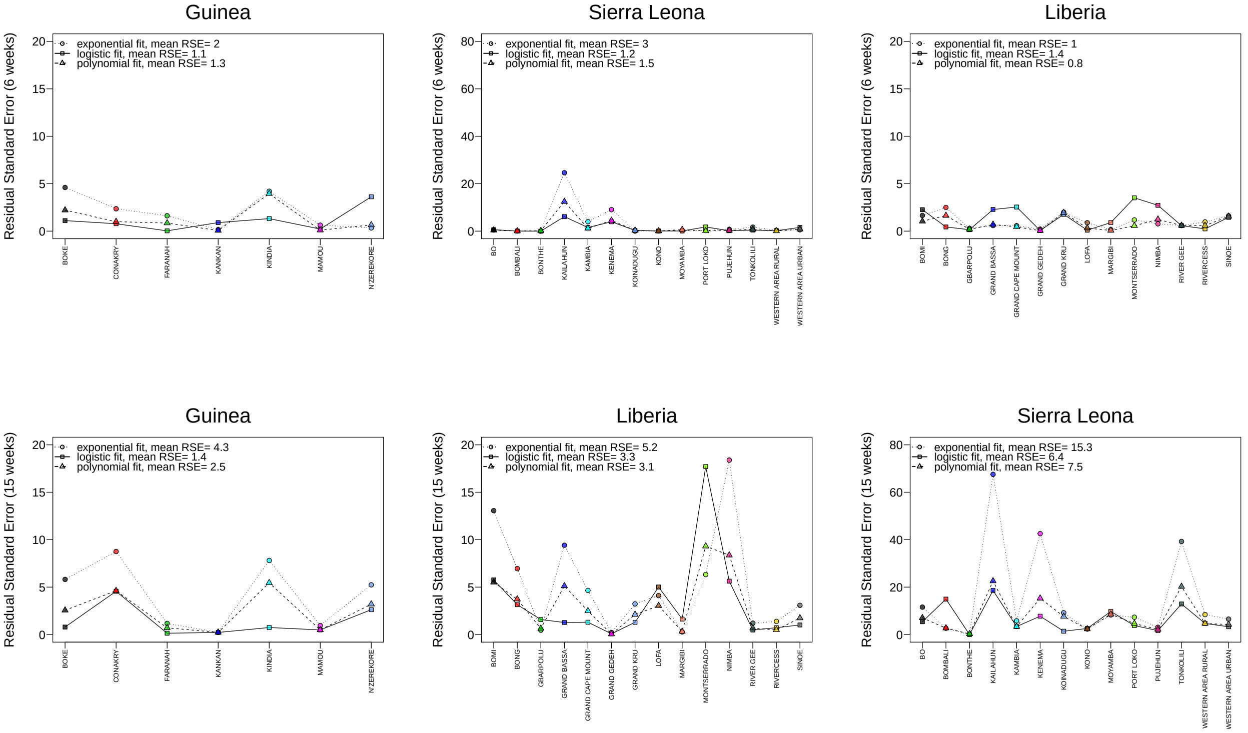

Figure S4. Comparing model Goodness of Fit in each regions, for each country, based on the residual standard error. Using data from the first 6 weeks (upper panel) and 15 weeks (lower panel) of the outbreak in each region.
