# Supplementary material for: Predicting Subnational Ebola Virus Disease Epidemic Dynamics from Sociodemographic Indicators
Source: PLoS One. 2016 Oct 12;11(10):e0163544. doi: 10.1371/journal.pone.0163544 (PMC5061396; doi:10.1371/journal.pone.0163544)
Supplement: S5 Fig — (PDF) [file pone.0163544.s005.pdf]

Predicting subnational Ebola virus disease epidemic dynamics from sociodemographic indicators

From Figure S5 we can easily conclude that, by ample margin, the estimate of m in the polynomial fit has, on average, the largest uncertainty of the three growth rates. In contrast, the exponential rate estimates r_e feature the lowest uncertainty, with the logistic growth estimates faring close to exponential ones (except for some cases (e.g., Port Loko) where the standard deviation is very large).

As expected, increasing the number of data points from 6 to 15 reduced, overall, the standard deviation of the respective rates.

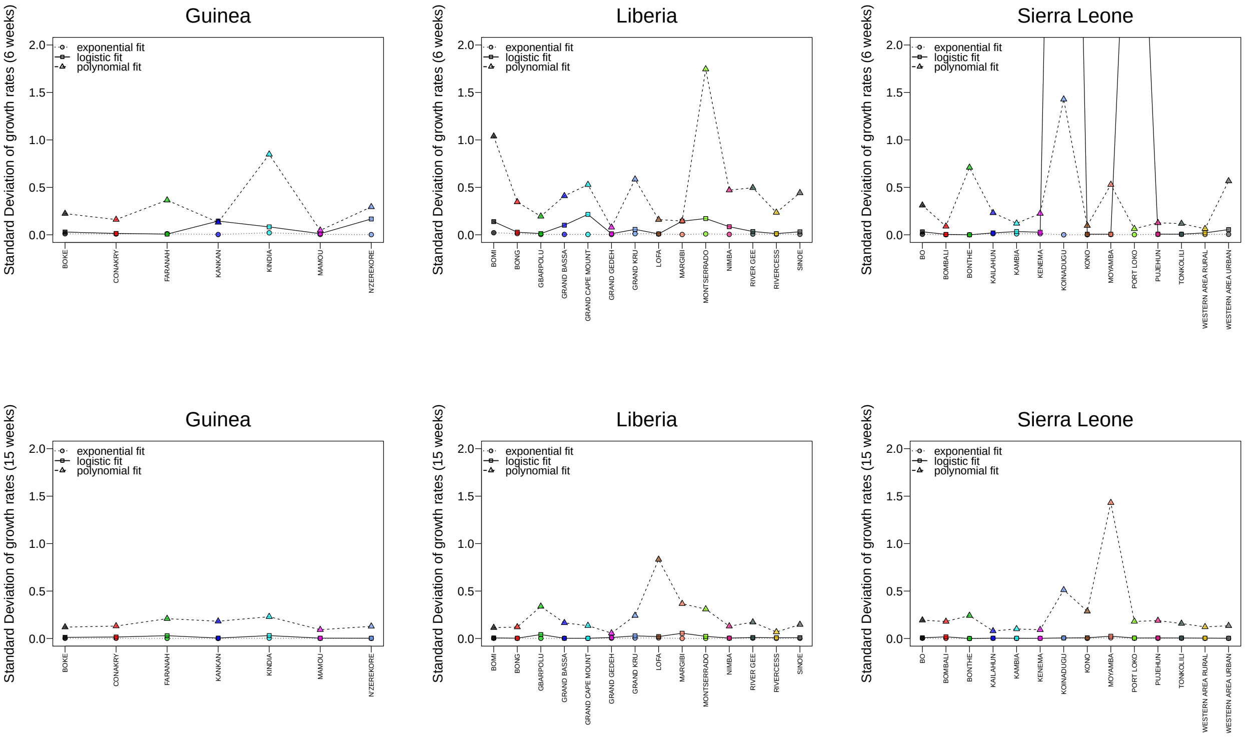

Figure S5. Comparison of standard deviation of growth rates estimates at different regions, and for each country. Considering the first 6 weeks (upper panel) and 15 weeks (lower panel) of the outbreaks in each region.
