# Supplementary material for: Predicting Subnational Ebola Virus Disease Epidemic Dynamics from Sociodemographic Indicators
Source: PLoS One. 2016 Oct 12;11(10):e0163544. doi: 10.1371/journal.pone.0163544 (PMC5061396; doi:10.1371/journal.pone.0163544)
Supplement: S6 Fig — (PDF) [file pone.0163544.s006.pdf]

Predicting subnational Ebola virus disease epidemic dynamics from sociodemographic indicators

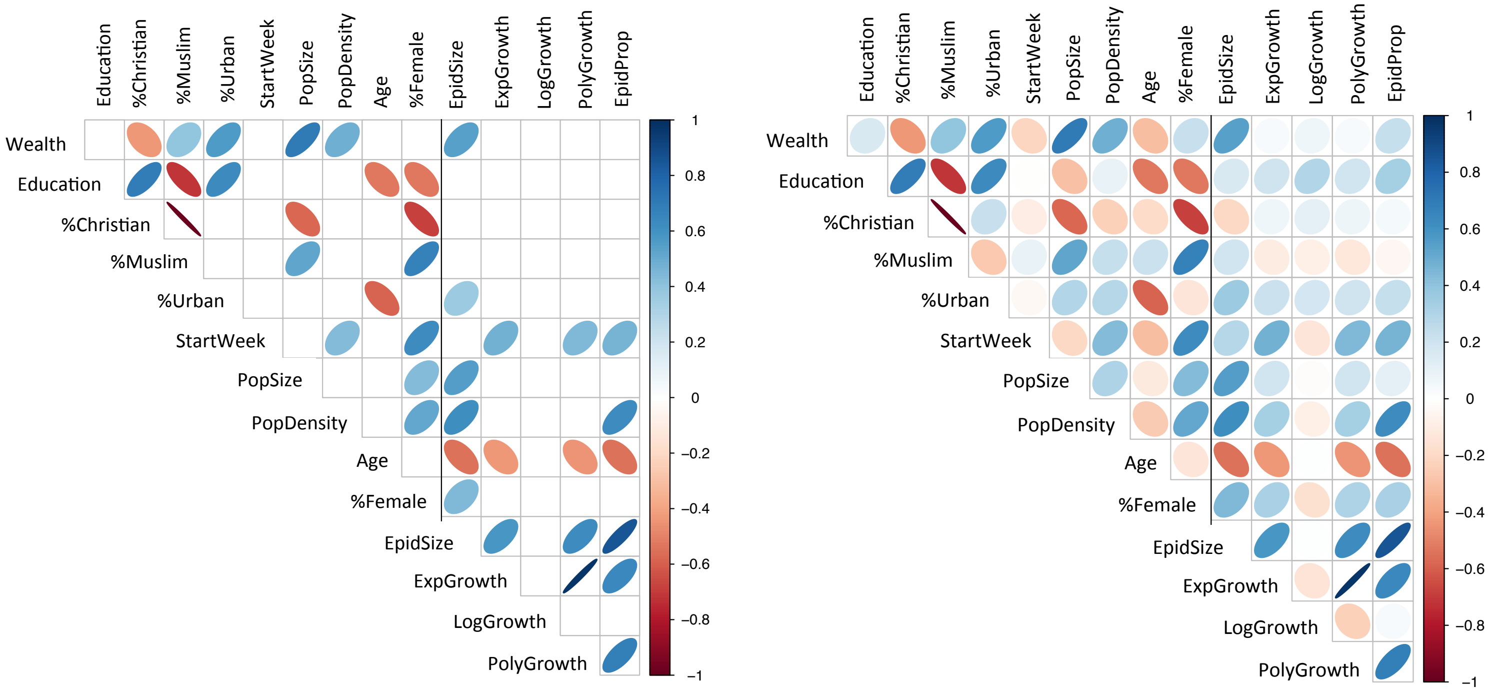

Figure S6. Pairwise Spearman correlation plots between outcomes and covariates. The direction of correlation is indicated by color type (blue: positive; red: negative). The strength of correlation is indicated by color intensity; uncertainty around the estimates is indicated by width of the ovals (wider: more uncertainty). In the first subplot we show only correlations statistically significant at $\alpha = 0.05$. Outcomes: regional growth rate estimated using data from first 15 weeks of epidemic in each region (exponential fit, "*ExpGrowth*"; logistic fit, "*LogGrowth*"; polynomial fit, "*PolyGrowth*"); total number of infections reported in each region ("*EpidSize*"), proportion of whole population infected ("*EpidProp*"). Socio-economic covariates: wealth index, "$Wealth$"; average years of education, "*Education*"; percent of population Christian, "$\%Christian$"; percent of population Muslim, "$\%Muslim$"; percent of population living in urban area, "$\%Urban$"; number of weeks from start of EVD in West Africa to the first recorded case in each region, "$StartWeek$"; population size, "*PopSize*"; population density, "*PopDensity*"; average age, "*Age*"; percent of population female, "$\%Female$").
