# Supplementary material for: Predicting Subnational Ebola Virus Disease Epidemic Dynamics from Sociodemographic Indicators
Source: PLoS One. 2016 Oct 12;11(10):e0163544. doi: 10.1371/journal.pone.0163544 (PMC5061396; doi:10.1371/journal.pone.0163544)
Supplement: S1 Table — (PDF) [file pone.0163544.s007.pdf]

Predicting subnational Ebola virus disease epidemic dynamics from sociodemographic indicators

Table S1. Descriptive statistics for growth rates estimates (exponential fit, logistic fit, polynomial fit), final epidemic sizes and socio-economic covariates at the regional level.

	Mean	S.D.	range
Wealth Index (iwi)	24.7	11.1	(13.7 - 69.8)
Average number of years of education	3.72	1.66	(1.50 - 8.20)
Proportion Christian	0.43	0.36	(0.01 - 0.961)
Proportion Muslim	0.54	0.38	(0.01 - 0.99)
Urbanicity (% of respondents in urban sampling units)	31.0	25.0	(4.87 - 100)
Week from start of EVD epidemic	12.4	7.4	(0 - 20)
Population size	487,715	479,768	(57,913 - 1,936,329)
Population density (per km^2)	4,852	25,864	(22 - 154,283)
Average age	29.9	1.15	(27.7 - 32.9)
% Female	48.0	0.10	(31.9 - 63.0)
Final Epidemic size	583	777.4	(2.00 - 3,146)
Exponential growth rate	0.04	0.02	(0.00 - 0.14)
Logistic growth rate	0.15	0.33	(0.00 - 2.00)
Polynomial growth rate	2.00	2.19	(0.05 - 11.78)
Final Epidemic percent (% of residents infected)	0.014	0.188	(0.001 - 0.985)

Note: Epidemic growth rates based on first 15 weeks of cases in each ADM2.
