# Supplementary material for: Predicting Subnational Ebola Virus Disease Epidemic Dynamics from Sociodemographic Indicators
Source: PLoS One. 2016 Oct 12;11(10):e0163544. doi: 10.1371/journal.pone.0163544 (PMC5061396; doi:10.1371/journal.pone.0163544)
Supplement: S2 Table — (PDF) [file pone.0163544.s008.pdf]

Predicting subnational Ebola virus disease epidemic dynamics from sociodemographic indicators

Table S2. Multivariable linear regression model for growth rates from exponential, logistic and polynomial models (6 weeks of data) and final epidemic size, adjusting for all predictors selected by step-wise backwards regression using the BIC criterion and further adjusting for country.

	Dependent variable:				
	exponential growth	logistic growth	polynomial growth	epidemic size	epidemic proportion
	(1)	(2)	(3)	(4)	(5)
Wealth index				-43.7^{*}<br>(−88.7, 1.21)	
Mean education (years)	0.01^{*}<br>(0.000, 0.01)		1.02^{***}<br>(0.34, 1.70)	454^{***}<br>(162, 746)	0.06^{***}<br>(0.02, 0.09)
Percent urban			-0.05^{**}<br>(−0.09, −0.004)		
Population (thousands)			0.002^{*}<br>(−0.000, 0.004)	0.97^{**}<br>(0.26, 1.67)	
Population density (per km^2)				0.01^{**}<br>(0.001, 0.02)	
Percent female			0.25^{**}<br>(0.07, 0.43)	67.2^{*}<br>(−1.52, 136)	0.01<br>(−0.01, 0.03)
Liberia	−0.01<br>(−0.03, 0.02)	0.04<br>(−0.26, 0.34)	3.15^{**}<br>(0.41, 5.90)	169<br>(−1,128, 1,468)	0.07<br>(−0.19, 0.33)
Sierra Leone	−0.002<br>(−0.03, 0.02)	−0.12<br>(−0.42, 0.18)	−2.00<br>(−5.17, 1.16)	−341<br>(−1,674, 992)	0.06<br>(−0.28, 0.39)
Constant	0.03^{*}<br>(−0.001, 0.05)	0.20<br>(−0.04, 0.45)	-14.14^{***}<br>(−23.18, −5.09)	$-3{,}699^{**}$<br>(−6,963, −434)	−0.57<br>(−1.66, 0.53)
Observations	35	35	35	35	35
Adjusted R^2	0.03	−0.01	0.33	0.62	0.27
Residual Std. Error	0.03	0.33	1.24	484.50	0.16
F Statistic	1.34	0.82	3.77^{***}	8.89^{***}	4.07^{***}

Note: Values are point estimates and 95% confidence intervals. $^{*}p<0.1$; $^{**}p<0.05$; $^{***}p<0.01$. F statistic is from a test that all coefficient values are jointly equal to zero. Outcome units: equations (1-2) 1/time; (3) unitless; (4) count number of reported cases; (5) percentage points of population reported as cases. Covariate units: education, one additional year; percent female, ADM2 is one additional percentage point female.
