# Supplementary material for: Predicting Subnational Ebola Virus Disease Epidemic Dynamics from Sociodemographic Indicators
Source: PLoS One. 2016 Oct 12;11(10):e0163544. doi: 10.1371/journal.pone.0163544 (PMC5061396; doi:10.1371/journal.pone.0163544)
Supplement: S1 Text — (DOCX) [file pone.0163544.s009.docx]

**S1 Text.** Stata and R code required to replicate results shown in this paper

**Title**: Predicting subnational Ebola virus disease epidemic dynamics from sociodemographic indicators

**Authors**: Linda Valeri, Oscar Patterson-Lomba, Yared Gurmu, Akweley Ablorh, Jennifer Bobb, William Townes, Guy Harling

The data for this paper came from several sources.

1. The Ebola time series data were downloaded from <https://data.hdx.rwlabs.org/dataset/evd-cases-by-district> and are provided in S1 File.
2. The International Wealth Index data were downloaded from <http://globaldatalab.org/iwi/> and are provided in S1 File.
3. The census population data were extracted from the three censuses referenced in the main text and are provided in S1 File.
4. The Demographic and Health Survey (DHS) data must be requested directly from DHS by following the instructions at <http://dhsprogram.com/data/Access-Instructions.cfm>. Running the code in section 1 below will provide the data in a form that can be used in the other code in this document.

There are six code pieces used in this analysis, covered in the six sections below:

1. Import DHS data for all countries and form it into a single file.
2. Reorganize the raw WHO Ebola data series into a useful format.
3. Generate and describe the Ebola growth curves for each region or district.
4. Tidy the DHS data and merge it with wealth and population data, and with Ebola curve summary statistics.
5. Describe the area-level covariates used in statistical analysis.
6. Conduct the area-level statistical analysis.

Section 1. Import DHS data for all countries and form it into a single file. Code written in Stata version 13

*** Set your working directory, where raw datasets are sitting in 3 sub-folders: IR (female); MR (male); PR (household)

*** We also use a fourth sub-directory for all generated datasets: Output

cap cd // working dir goes here

set more off

local filelist_ir "LBIR6AFL SLIR61FL GNIR61FL MLIR6HFL SNIR61FL CIIR61FL"

local filelist_mr "LBMR6AFL SLMR61FL GNMR61FL MLMR6HFL SNMR61FL CIMR61FL"

local filelist_pr "LBPR6AFL SLPR61FL GNPR61FL MLPR6HFL SNPR61FL CIPR61FL"

local country_list "Liberia SierraLeone Guinea Mali Senegal CoteDIvoire"

local region_var "scounty sdist v024 v024 v024 v024"

local region_var_m "smcounty smdist mv024 mv024 mv024 mv024"

*** This pulls in all the Female interviews

forvalues i = 1/6 {

if `i' == 1 {

local tot_pop = 919098

}

if `i' == 2 {

local tot_pop = 1379234

}

if `i' == 3 {

local tot_pop = 2525223

}

if `i' == 4 {

local tot_pop = 3328267

}

if `i' == 5 {

local tot_pop = 3153450

}

if `i' == 6 {

local tot_pop = 4565824

}

local f : word `i' of `filelist_ir'

local c : word `i' of `country_list'

use v* s* using "IR/`f'.dta", clear

gen country = `i'

gen double unique_id = `i' * 100000000 + v001 * 1000000 + v002 * 1000 + v003

* age - v012

gen female = 1

* region - these vary, so specify in local above

local region : word `i' of `region_var'

decode `region', gen(regions)

* urban - v025

* education - v106

* wealth - v190

* toilet type - v116

decode v116, gen(toilets)

* religion - v130

decode v130, gen(religions)

* ethnicity - v131: careful, includes dialect sometimes (eg Liberia)

decode v131, gen(ethnicitys)

* mobility - v167/8 - missing for Liberia; v168 requires >0 for v167

* radio in hhold - v120

* freq listen to radio - v158

* transport: bike: v123; motorbike: v124; car: v125

* sampling weight - v005

local fem_iv_tot = _N

local fem_sf = `fem_iv_tot' / `tot_pop'

gen v005a = v005 / `fem_sf' / 1000000

replace v005 = v005 / 1000000

keep unique_id country v001-v003 v005* female v012 ///

regions toilets religions ethnicitys ///

v025 v106 v120 v123-v125 ///

v158 v167 v168 v190

saveold `"Output/`f'.dta"', replace

}

*** Quick detour to household dataset - men's data doesn't include household variables

forvalues i = 1/6 {

local f : word `i' of `filelist_pr'

use "PR/`f'.dta", clear

bys hv001 hv002: keep if _n == 1

foreach v in v001 v002 v003 {

rename h`v' `v'

}

rename hv205 v116

rename hv207 v120

rename hv210 v123

rename hv211 v124

rename hv212 v125

keep v*

saveold `"Output/`f'.dta"', replace

}

*** And then the men's dataset

forvalues i = 1/6 {

if `i' == 1 {

local tot_pop = 1478778

}

if `i' == 2 {

local tot_pop = 930023

}

if `i' == 3 {

local tot_pop = 2854343

}

if `i' == 4 {

local tot_pop = 3723573

}

if `i' == 5 {

local tot_pop = 3220525

}

if `i' == 6 {

local tot_pop = 5318884

}

local f : word `i' of `filelist_mr'

local p : word `i' of `filelist_pr'

local c : word `i' of `country_list'

cap use mv* s* using "MR/`f'.dta", clear

if `i' == 4 {

use mv* using "MR/`f'.dta", clear

}

gen country = `i'

gen double unique_id = `i' * 1000000000 + mv001 * 1000000 + mv002 * 1000 + mv003

gen female = 0

local region : word `i' of `region_var_m'

decode `region', gen(regions)

decode mv130, gen(religions)

decode mv131, gen(ethnicitys)

foreach v in v001 v002 v003 v005 v012 v025 v106 v158 v167 v168 v190 {

rename m`v' `v'

}

local mal_iv_tot = _N

local mal_sf = `mal_iv_tot' / `tot_pop'

gen v005a = v005 / `mal_sf' / 1000000

replace v005 = v005 / 1000000

keep unique_id country v001-v003 v005* female v012 regions regions religions ethnicitys ///

v025 v106 v158 v167 v168 v190

merge m:1 v001 v002 using `"HackEbola/`p'.dta"'

keep if _merge == 3

drop _merge

decode v116, gen(toilets)

drop v116

saveold `"Output/`f'.dta"', replace

}

*** Append the 12 datasets together

use "Output/LBIR6AFL.dta", clear

forvalues i = 2/6 {

local f : word `i' of `filelist_ir'

append using `"Output/`f'.dta"'

}

forvalues i = 1/6 {

local f : word `i' of `filelist_mr'

append using `"Output/`f'.dta"'

}

*** Labels, names, data tidying

order country unique_id, before(v001)

order female, before(v012)

gsort country v001 v002 -female v003

lab def sexf 0 "Male" 1 "Female"

lab val female sexf

lab var female "Female sex"

lab def yesnof 0 "No" 1 "Yes"

lab def cntryf 1 "Liberia" 2 "Sierra Leone" 3 "Guinea" 4 "Mali" 5 "Senegal" 6 "CoteDIvoire"

lab val country cntryf

local rename_vars v001 v002 v003 v005 v012 v025 v106 v120 v123 ///

v124 v125 v158 v167 v168 v190

local rename_names clust_no hhold_no line_no wgt_indiv age urban educat radio_own bicycle ///

motorbike car radio_listen trips12m away1m wealth_q

forvalues i = 1/15 {

local v : word `i' of `rename_vars'

local n : word `i' of `rename_names'

rename `v' `n'

}

* Shift variables back to numbers:

foreach x in region toilet religion ethnicity {

encode `x's, gen(`x')

drop `x's

}

lab var ethnicity "Ethnicity or Dialect"

replace religion = 1 if religion == 2 | religion == 17 | religion == 18

replace religion = 5 if religion == 6 | religion == 15

replace religion = 10 if religion == 8 | religion == 11

replace religion = 12 if religion == 13 | religion == 16

lab def religion 1 "Animist/Traditional" , modify

replace toilet = 6 if toilet == 7

replace toilet = . if toilet == 11 | toilet == 12

replace educat = . if educat == 9

foreach x in radio_own bicycle motorbike car {

replace `x' =. if `x' > 6

}

replace radio_listen = . if radio_listen == 9

lab var away1m "Away for more than one month in last 12 months"

lab var trips12m "Number of trips in last 12 months"

replace trips12m = 90 if trips12m > 89

replace away1m = . if away1m == 9

lab var ethnicity "Ethnicity or Dialect"

replace urban = 2 - urban

lab var urban "Place of residence is urban"

lab val urban yesnof

*** Generate a stratum variable, based on region and urbanicity

gen stratum = region * 2

replace stratum = stratum +1 if urban == 1

#delimit ;

lab def stratumf

2 "Bamako - rural"

3 "Bamako - urban"

4 "Bo - rural"

5 "Bo - urban"

6 "Boké - rural"

7 "Boké - urban"

8 "Bombali - rural"

9 "Bombali - urban"

10 "Bomi - rural"

11 "Bomi - urban"

12 "Bong - rural"

13 "Bong - urban"

14 "Bonthe - rural"

15 "Bonthe - urban"

16 "Centre - rural"

17 "Centre - urban"

18 "Centre-Est - rural"

19 "Centre-Est - urban"

20 "Centre-Nord - rural"

21 "Centre-Nord - urban"

22 "Centre-Ouest - rural"

23 "Centre-Ouest - urban"

24 "Conakry - rural"

25 "Conakry - urban"

26 "Dakar - rural"

27 "Dakar - urban"

28 "Diourbel - rural"

29 "Diourbel - urban"

30 "Faranah - rural"

31 "Faranah - urban"

32 "Fatick - rural"

33 "Fatick - urban"

34 "Gbarpolu - rural"

35 "Gbarpolu - urban"

36 "Grand Bassa - rural"

37 "Grand Bassa - urban"

38 "Grand Cape Mount - rural"

39 "Grand Cape Mount - urban"

40 "Grand Gedeh - rural"

41 "Grand Gedeh - urban"

42 "Grand Kru - rural"

43 "Grand Kru - urban"

44 "Kaffrine - rural"

45 "Kaffrine - urban"

46 "Kailahun - rural"

47 "Kailahun - urban"

48 "Kambia - rural"

49 "Kambia - urban"

50 "Kankan - rural"

51 "Kankan - urban"

52 "Kaolack - rural"

53 "Kaolack - urban"

54 "Kayes - rural"

55 "Kayes - urban"

56 "Kedougou - rural"

57 "Kedougou - urban"

58 "Kenema - rural"

59 "Kenema - urban"

60 "Kindia - rural"

61 "Kindia - urban"

62 "Koinadugu - rural"

63 "Koinadugu - urban"

64 "Kolda - rural"

65 "Kolda - urban"

66 "Kono - rural"

67 "Kono - urban"

68 "Koulikoro - rural"

69 "Koulikoro - urban"

70 "Labé - rural"

71 "Labé - urban"

72 "Lofa - rural"

73 "Lofa - urban"

74 "Louga - rural"

75 "Louga - urban"

76 "Mamou - rural"

77 "Mamou - urban"

78 "Margibi - rural"

79 "Margibi - urban"

80 "Maryland - rural"

81 "Maryland - urban"

82 "Matam - rural"

83 "Matam - urban"

84 "Montserrado - rural"

85 "Montserrado - urban"

86 "Mopti - rural"

87 "Mopti - urban"

88 "Moyamba - rural"

89 "Moyamba - urban"

90 "N'Zérékoré - rural"

91 "N'Zérékoré - urban"

92 "Nimba - rural"

93 "Nimba - urban"

94 "Nord - rural"

95 "Nord - urban"

96 "Nord-Ouest - rural"

97 "Nord-Ouest - urban"

98 "Nord-est - rural"

99 "Nord-est - urban"

100 "Ouest - rural"

101 "Ouest - urban"

102 "Port Loko - rural"

103 "Port Loko - urban"

104 "Pujehun - rural"

105 "Pujehun - urban"

106 "River Cess - rural"

107 "River Cess - urban"

108 "River Gee - rural"

109 "River Gee - urban"

110 "Saint-Louis - rural"

111 "Saint-Louis - urban"

112 "Sedhiou - rural"

113 "Sedhiou - urban"

114 "Segou - rural"

115 "Segou - urban"

116 "Sikasso - rural"

117 "Sikasso - urban"

118 "Sinoe - rural"

119 "Sinoe - urban"

120 "Sud sans Abidjan - rural"

121 "Sud sans Abidjan - urban"

122 "Sud-ouest - rural"

123 "Sud-ouest - urban"

124 "Tambacounda - rural"

125 "Tambacounda - urban"

126 "Thiès - rural"

127 "Thiès - urban"

128 "Tonkolili - rural"

129 "Tonkolili - urban"

130 "Ville d'Abidjan - rural"

131 "Ville d'Abidjan - urban"

132 "Western Rural - rural"

133 "Western Rural - urban"

134 "Western Urban - rural"

135 "Western Urban - urban"

136 "Ziguinchor - rural"

137 "Ziguinchor - urban" ;

#delimit cr

lab val stratum stratumf

lab var stratum "Sample stratum within country"

rename wgt_indiv wgt_sex_specifi

rename v005a wgt_indiv

*** Re-normalize the joint dataset

qui sum wgt_indiv

di `r(mean)'

replace wgt_indiv = wgt_indiv / `r(mean)'

saveold "Output/MergedDataset.dta", replace

Section 2. Reorganize the raw WHO Ebola data series into a useful format. Code written in R version 3.2

##################

###Code for processing DATA from WHO website(Number of Cases)

# Weekly report of the cases obtained from WHO website:

#http://apps.who.int/gho/data/node.ebola-sitrep.quick-downloads?lang=en)

##

# READ IN RAW TIME SERIES DATA (CASES UP TO 7/29/2015)

#Raw time-series data from Liberia is saved as liberia.cases

ebola.l<-read.csv(file="liberia.cases.csv",head=TRUE,sep=",")

#Raw time-series data from Guinea is saved as guinea.cases

ebola.g<-read.csv(file="guinea.cases.csv",head=TRUE,sep=",")

#Raw time-series data from Sierra Leone is saved as sierraleone.cases

ebola.s<-read.csv(file="sierraleone.cases.csv",head=TRUE,sep=",")

#2. Create key to merge by

ebola.l$reg.id<-ebola.l$Location

ebola.s$reg.id<-ebola.s$Location

ebola.g$reg.id<-ebola.g$Location

########################### LIBERIA DATA############################################

#Merge by region name: Liberia

ebola.l<-ebola.l[!is.na(ebola.l$Numeric),] # deleting all rows with no entries for cases

ebola.l<-subset(ebola.l,Ebola.data.source=="Patient database") # Note: Restricted to patient database only(in-country reporting)

datec<-list(NULL) # list of the dates

dates<-strsplit(as.character(ebola.l$Epi.week)," ")

for (i in 1:dim(ebola.l)[1]){

##Depending on the date format AND LENGTH, we need to adjust the values extracted

if(length(dates[[i]])==6){

datec[[i]]<-(dates[[i]][c(3,4,5)]) #pick only the End-date, Month, Year

}else if(length(dates[[i]])==7){

datec[[i]]<-(dates[[i]][c(4,5,6)])

}else{

datec[[i]]<-(dates[[i]][c(5,6,7)])

}

ebola.l$dates[i]<-as.character(as.Date(paste(datec[[i]][1],datec[[i]][2],datec[[i]][3]), format('%d %B %Y'))) # convert to R-dates

}

####Unaggregated

ebola.lunagg<-data.frame(reg.id=ebola.l$reg.id,dates=ebola.l$dates,Numeric=ebola.l$Numeric)

ebola.lunagg<-ebola.lunagg[(ebola.lunagg$reg.id!=""),]

ebola.lunagg<-ebola.lunagg[order(ebola.lunagg$reg.id,ebola.lunagg$dates),]

startdata<-data.frame(reg.id=unique(ebola.lunagg$reg.id),start.date=(ebola.lunagg$date[!duplicated(ebola.lunagg$reg.id)]))

ebola.lunagg<-merge(ebola.lunagg,startdata,by="reg.id")

ebola.lunagg$weekstart<-round(as.numeric(difftime(strptime(ebola.lunagg$start.date, format = "%Y-%m-%d"),

strptime("2014-01-05", format = "%Y-%m-%d"),units="weeks")))

####Aggregated

ebola.lagg<-as.data.frame(aggregate(ebola.lunagg$Numeric~ebola.lunagg$reg.id,ebola.lunagg ,FUN= "sum"))

ebola.lagg$weekstart<-ebola.lunagg$weekstart[!duplicated(ebola.lunagg$reg.id)]

names(ebola.lagg)<-c("reg.id","Numeric","weekstart")##

## Aggregated across weeks

write.csv(ebola.lagg,file="liberia.aggregated_july.csv")

## Liberia unaggregated data

write.csv(ebola.lunagg,file="liberia.unaggregated_july.csv")

########################### GUINEA DATA############################################

##Remove missing region name

ebola.g<-ebola.g[!is.na(ebola.g$Numeric),] # deleting all rows with no entries for cases

ebola.g<-subset(ebola.g,Ebola.data.source=="Patient database") # Note: Restricted to patient database only(in-country reporting)

datec<-list(NULL) # list of the dates

dates<-strsplit(as.character(ebola.g$Epi.week)," ")

for (i in 1:dim(ebola.g)[1]){

##Depending on the date format AND LENGTH, we need to adjust the values extracted

if(length(dates[[i]])==6){

datec[[i]]<-(dates[[i]][c(3,4,5)]) #pick only the End-date, Month, Year

}else if(length(dates[[i]])==7){

datec[[i]]<-(dates[[i]][c(4,5,6)])

}else{

datec[[i]]<-(dates[[i]][c(5,6,7)])

} #pick only the End-date, Month, Year

ebola.g$dates[i]<-as.character(as.Date(paste(datec[[i]][1],datec[[i]][2],datec[[i]][3]), format('%d %B %Y'))) # convert to R-dates

}

## Only important variables are kept from HDX dataset: Number of cases(Numeric), Region ID (reg.id), Dates(dates), EPI_WEEK..DISPLAY.

ebola.gagg<-ebola.g[,c("reg.id","Numeric","dates")]

## NOTE: Need to collapse by region and week since covariate information is only available at the region level(ADM1 level)

ebola.g$state<-ifelse(ebola.g$reg.id %in% c("FRIA","BOKE","BOFFA"),"BOKE",

ifelse(ebola.g$reg.id %in% c("CONAKRY"),"CONAKRY",

ifelse(ebola.g$reg.id %in% c("DABOLA","DINGUIRAYE","KISSIDOUGO","FARANAH"),"FARANAH",

ifelse(ebola.g$reg.id %in% c("KANKAN","KOUROUSSA","SIGUIRI","KEROUANE"),"KANKAN",

ifelse(ebola.g$reg.id %in% c("COYAH","TELIMELE","FORECARIAH","DUBREKA","KINDIA"),"KINDIA",

ifelse(ebola.g$reg.id %in% c("DALABA","PITA"),"MAMOU",

ifelse(ebola.g$reg.id %in% c("MACENTA","LOLA","YOMOU","BEYLA","GUECKEDOU","N'ZEREKORE"),"N'ZEREKORE","NONE"

)

)

)

)

)

)

)

####Unaggregated

#ebola.g<-ebola.g[,-c(ebola.g$reg.id)]

ebola.gunagg<-data.frame(reg.id=ebola.g$state,dates=ebola.g$dates,Numeric=ebola.g$Numeric)

ebola.gunagg<-ebola.gunagg[(ebola.gunagg$reg.id!=""),]

ebola.gunagg<-ebola.gunagg[order(ebola.gunagg$reg.id,ebola.gunagg$dates),]

startdata<-data.frame(reg.id=unique(ebola.gunagg$reg.id),start.date=(ebola.gunagg$date[!duplicated(ebola.gunagg$reg.id)]))

ebola.gunagg<-merge(ebola.gunagg,startdata,by="reg.id")

ebola.gunagg$weekstart<-round(as.numeric(difftime(strptime(ebola.gunagg$start.date, format = "%Y-%m-%d"),

strptime("2014-01-05", format = "%Y-%m-%d"),units="weeks")))

####Aggregated

ebola.gagg<-as.data.frame(aggregate(ebola.gunagg$Numeric~ebola.gunagg$reg.id,ebola.gunagg ,FUN= "sum"))

ebola.gagg$weekstart<-ebola.gunagg$weekstart[!duplicated(ebola.gunagg$reg.id)]

names(ebola.gagg)<-c("reg.id","Numeric","weekstart")##

## Aggregated across weeks

write.csv(ebola.gagg,file="guinea.aggregated_july.csv")

## Unaggregated data

write.csv(ebola.gunagg,file="guinea.unaggregated_july.csv")

########################### SIERRALIONE DATA############################################

##Remove missing region name

ebola.s<-ebola.s[!is.na(ebola.s$Numeric),] # deleting all rows with no entries for cases

ebola.s<-subset(ebola.s,Ebola.data.source=="Patient database")

datec<-list(NULL) # list of the dates

dates<-strsplit(as.character(ebola.s$Epi.week)," ")

for (i in 1:dim(ebola.s)[1]){

##Depending on the date format AND LENGTH, we need to adjust the values extracted

if(length(dates[[i]])==6){

datec[[i]]<-(dates[[i]][c(3,4,5)]) #pick only the End-date, Month, Year

}else if(length(dates[[i]])==7){

datec[[i]]<-(dates[[i]][c(4,5,6)])

}else{

datec[[i]]<-(dates[[i]][c(5,6,7)])

} #pick only the End-date, Month, Year

ebola.s$dates[i]<-as.character(as.Date(paste(datec[[i]][1],datec[[i]][2],datec[[i]][3]), format('%d %B %Y'))) # convert to R-dates

}

####Unaggregated

ebola.sunagg<-data.frame(reg.id=ebola.s$reg.id,dates=ebola.s$dates,Numeric=ebola.s$Numeric)

ebola.sunagg<-ebola.sunagg[(ebola.sunagg$reg.id!=""),]

ebola.sunagg<-ebola.sunagg[order(ebola.sunagg$reg.id,ebola.sunagg$dates),]

startdata<-data.frame(reg.id=unique(ebola.sunagg$reg.id),start.date=(ebola.sunagg$date[!duplicated(ebola.sunagg$reg.id)]))

ebola.sunagg<-merge(ebola.sunagg,startdata,by="reg.id")

ebola.sunagg$weekstart<-round(as.numeric(difftime(strptime(ebola.sunagg$start.date, format = "%Y-%m-%d"),

strptime("2014-01-05", format = "%Y-%m-%d"),units="weeks")))

####Aggregated

ebola.sagg<-as.data.frame(aggregate(ebola.sunagg$Numeric~ebola.sunagg$reg.id,ebola.sunagg ,FUN= "sum"))

ebola.sagg$weekstart<-ebola.sunagg$weekstart[!duplicated(ebola.sunagg$reg.id)]

names(ebola.sagg)<-c("reg.id","Numeric","weekstart")##

## Aggregated across weeks

write.csv(ebola.sagg,file="sierra.aggregated_july.csv")

## Sierra unaggregated data

write.csv(ebola.sunagg,file="sierra.unaggregated_july.csv")

Section 3. Generate and describe the Ebola growth curves for each region or district. Code written in R version 3.2

cat("\014")

rm(list = ls())

if(!"nls2"%in%.packages(all=TRUE)) (install.packages("nls2"))

if(!"lubridate"%in%.packages(all=TRUE)) (install.packages("lubridate"))

if(!"chron"%in%.packages(all=TRUE)) (install.packages("chron"))

if(!"sfsmisc"%in%.packages(all=TRUE)) (install.packages("sfsmisc"))

if(!"scales"%in%.packages(all=TRUE)) (install.packages("scales"))

if(!"plyr"%in%.packages(all=TRUE)) (install.packages("plyr"))

require(nls2)

require(lubridate)

require(chron)

require(sfsmisc)

require(scales)

require(plyr)

##################################################

# 1. Read in the data and tidy

guinea=read.csv("guinea.unaggregated_july.csv")

#eliminating entries with no reports

if(length(which(guinea$Numeric==0))>0) guinea=guinea[-which(guinea$Numeric==0),]

agg_guinea=aggregate(Numeric ~ dates+reg.id, data = guinea, sum)

agg_guinea <- agg_guinea[order(as.Date(agg_guinea$dates)),] # ordering by date

guinea1=agg_guinea

## adding dates as days since beginign of reporting

guinea1$dates21=NA

guinea1$dates21=julian(month(guinea1$dates),day(guinea1$dates),year(guinea1$dates))

guinea1$dates22=NA

# deleting NONE

guinea1=guinea1[-which(guinea1$reg.id=="NONE"),]

guinea1$reg.id <- factor(guinea1$reg.id)

sierra=read.csv("sierra.unaggregated_july.csv")

#eliminating entries with no reports

if(length(which(sierra$Numeric==0))>0) sierra=sierra[-which(sierra$Numeric==0),]

agg_sierra=aggregate(Numeric ~ dates+reg.id, data = sierra, sum)

agg_sierra <- agg_sierra[order(as.Date(agg_sierra$dates)),] # ordering by date

sierra1=agg_sierra

## adding dates as days since beginign of reporting

sierra1$dates21=NA

sierra1$dates21=julian(month(sierra1$dates),day(sierra1$dates),year(sierra1$dates))

sierra1$dates22=NA

liberia=read.csv("liberia.unaggregated_july.csv")

#eliminating entries with no reports

if(length(which(liberia$Numeric==0))>0) liberia=liberia[-which(liberia$Numeric==0),]

agg_liberia=aggregate(Numeric ~ dates+reg.id, data = liberia, sum)

agg_liberia <- agg_liberia[order(as.Date(agg_liberia$dates)),] # ordering by date

liberia1=agg_liberia

## adding dates as days since beginign of reporting

liberia1$dates21=NA

liberia1$dates21=julian(month(liberia1$dates),day(liberia1$dates),year(liberia1$dates))

liberia1$dates22=NA

# deleting Maryland due to lack of data

liberia1=liberia1[-which(liberia1$reg.id=="MARYLAND"),]

liberia1$reg.id <- factor(liberia1$reg.id)

### first time of an entry at start of reporting

tmin0=min(c(guinea1$dates21,liberia1$dates21,sierra1$dates21))

guinea1$dates22=guinea1$dates21-tmin0+1

sierra1$dates22=sierra1$dates21-tmin0+1

liberia1$dates22=liberia1$dates21-tmin0+1

tmax0=max(c(guinea1$dates22,liberia1$dates22,sierra1$dates22))

col_vec=c(1:6,'cornflowerblue','chocolate4','coral1','chartreuse1','deeppink2','darkslategray','gold2','gray35','darkred')

date_labs=c("Jan 2014", "Apr 2014","Jul 2014","Oct 2014","Jan 2015","Apr 2015","Jul 2015")

##################################################

# 2. Plot the raw cummulative case data

### Build plot with three panels

# cairo_pdf("Fig1_v5.tif",width=7.5, height=2.5,bg="transparent")

tiff("Fig1_v5.tiff", units="in", width=7.5, height=2.5, pointsize = 12,res=350)

mult.fig(mfrow=c(1,3))

par(oma=c(1,1,1,1))

par(mai=c(.3,.5,.3,0))

# Guinea

plot(unique(guinea1$dates22), rep(-1000,length(unique(guinea1$dates22))),

xlab="", ylab="", ylim=c(0,3100), xlim=c(0,570),

cex.axis=0.9, cex=1.2,las=1, yaxt='n', xaxt='n', ann=FALSE)

axis(1, at=seq(0,600,by=90), lab=F, cex.axis=0.9,tck=-0.01,mgp=c(3,1,0))

text(seq(0,600,by=90),-250, labels = date_labs, srt = 45, pos = 1, xpd = TRUE,cex=0.75)

axis(2, at=seq(0,3000,by=300), lab=seq(0,3000,by=300),cex.axis=0.75, las=2,tck=-0.01)

mtext("Cumulative Cases", side=2, line=3,cex=0.6)

mtext("A. Guinea", side=3, line=1,cex=0.8)

glev=levels(guinea1$reg.id)

v1=1:length(glev)

for (i in v1){

sub_data=subset(guinea1,reg.id==glev[i])

cases=sub_data$Numeric

case_data=cumsum(cases)

times=as.numeric(sub_data$dates22)

deltaT=7 ### time in between reports

while(length(which(diff(times)>deltaT))>0){

donde=which(diff(times)>deltaT)[1] # where in the vector of diff is it missing

cuando=times[donde] # what day

cuanto=diff(times)[donde] # how many days are missing

inser=cuanto/deltaT-1 # number of data points (weeks) to insert

new_cases=rep(NA,inser)

new_times=rep(NA,inser)

slope=(case_data[donde+1]-case_data[donde])/(cuanto)

interc=case_data[donde]

for (np in 1:inser){

new_cases[np]=deltaT*np*slope+interc

new_times[np]=cuando+deltaT*np

}

case_data=c(case_data[1:donde],new_cases,case_data[(donde+1):length(case_data)])

times=c(times[1:donde],new_times,times[(donde+1):length(times)])

} # end of while

case_data=cbind(case_data, times)

colnames(case_data)=c("cumcases","time")

case_data1=as.data.frame(case_data)

max_cases=tail(cumsum(cases),1)

min_time=min(as.numeric(sub_data$dates22))

case_data2=cbind(case_data1,case_data1$time-min_time+1 )

colnames(case_data2)=c("cumcases","time","time2")

case_data2=as.data.frame(case_data2)

# adding the flat tail at the "end" of the epidemic (up to July 2015) where no new cases were reported

inser2=(tmax0-max(case_data2$time))/deltaT-1

if(inser2>0){

case_data2_tail=as.data.frame(cbind(rep(max_cases,inser2+1),seq(max(case_data2$time)+deltaT,tmax0,deltaT),rep(NA,inser2+1)))

names(case_data2_tail)=c("cumcases","time","time2")

case_data2=rbind(case_data2,case_data2_tail)

}

points(case_data2$time,case_data2$cumcases, col="dimgray",bg=alpha(col_vec[i],0.5), pch=21,lwd=0.5, cex=0.4)

lines(case_data2$time,case_data2$cumcases, col=alpha(col_vec[i],0.5),lwd=2,lty=1)

legend(x=1,3200-(i-1)*130,legend=glev[i],col=alpha(col_vec[i],0.7),lwd=2.5, cex=0.6,bty="n")

}

### Sierra Leone

plot(unique(sierra1$dates22), rep(-1000,length(unique(sierra1$dates22))),

xlab="", ylab="",ylim=c(0,3100),xlim=c(0,570),

cex.axis=0.9, cex=1.2,las=1, yaxt='n', xaxt='n', ann=FALSE)

axis(1, at=seq(0,600,by=90), lab=F, cex.axis=0.9,tck=-0.01,mgp=c(3,1,0))

text(seq(0,600,by=90),-250, labels = date_labs, srt = 45, pos = 1, xpd = TRUE,cex=0.75)

axis(2, at=seq(0,3000,by=300), lab=seq(0,3000,by=300),cex.axis=0.75, las=2,tck=-0.01)

#mtext("Cumulative Cases", side=2, line=3,cex=1)

mtext("B. Sierra Leone", side=3, line=1,cex=0.8)

glev=levels(sierra1$reg.id)

gleva = glev

gleva[c(13,14)]=c("WEST. AREA RURAL","WEST. AREA URBAN") # for legend

params=matrix(NA,length(glev),15) # c(region,cexp,rexp,rexp_se,rstexp,c1poly,c2poly,mpoly,mpoly_se,rstpoly,klogi,clogi,rlogi,rlogi_se,rstlogi)

maxweek=1000

inc_all_sierra=rep(0,600)

v1=1:length(glev)

for (i in v1){

sub_data=subset(sierra1,reg.id==glev[i])

cases=sub_data$Numeric

#if(length(cases)>5){

case_data=cumsum(cases)

times=as.numeric(sub_data$dates22)

#plot(times,case_data)

deltaT=7 ### time in between reports

while(length(which(diff(times)>deltaT))>0){

donde=which(diff(times)>deltaT)[1] # where in the vector of diff is it missing

cuando=times[donde] # what day

cuanto=diff(times)[donde] # how many days are missing

inser=cuanto/deltaT-1 # number of data points (weeks) to insert

new_cases=rep(NA,inser)

new_times=rep(NA,inser)

slope=(case_data[donde+1]-case_data[donde])/(cuanto)

interc=case_data[donde]

for (np in 1:inser){

new_cases[np]=deltaT*np*slope+interc

new_times[np]=cuando+deltaT*np

}

case_data=c(case_data[1:donde],new_cases,case_data[(donde+1):length(case_data)])

times=c(times[1:donde],new_times,times[(donde+1):length(times)])

} # end of while

case_data=cbind(case_data, times)

colnames(case_data)=c("cumcases","time")

case_data1=as.data.frame(case_data)

max_cases=tail(cumsum(cases),1)

min_time=min(as.numeric(sub_data$dates22))

case_data2=cbind(case_data1,case_data1$time-min_time+1 )

colnames(case_data2)=c("cumcases","time","time2")

case_data2=as.data.frame(case_data2)

# adding the flat tail at the "end" of the epidemic (up to July 2015) where no new cases were reported

inser2=(tmax0-max(case_data2$time))/deltaT-1

if(inser2>0){

case_data2_tail=as.data.frame(cbind(rep(max_cases,inser2+1),seq(max(case_data2$time)+deltaT,tmax0,deltaT),rep(NA,inser2+1)))

names(case_data2_tail)=c("cumcases","time","time2")

case_data2=rbind(case_data2,case_data2_tail)

}

points(case_data2$time,case_data2$cumcases, col="dimgray",bg=alpha(col_vec[i],0.5), pch=21,lwd=0.5, cex=0.4)

lines(case_data2$time,case_data2$cumcases, col=alpha(col_vec[i],0.5),lwd=2,lty=1)

legend(x=1,3200-(i-1)*130,legend=gleva[i],col=alpha(col_vec[i],0.7),lwd=2.5, cex=0.6,bty="n")

}

### Liberia

plot(unique(liberia1$dates22), rep(-1000,length(unique(liberia1$dates22))),

xlab="", ylab="",ylim=c(0,3100),xlim=c(0,570),

cex.axis=0.9, cex=1.2,las=1, yaxt='n', xaxt='n', ann=FALSE)

axis(1, at=seq(0,600,by=90), lab=F, cex.axis=0.9,tck=-0.01,mgp=c(3,1,0))

text(seq(0,600,by=90),-250, labels = date_labs, srt = 45, pos = 1, xpd = TRUE,cex=0.75)

axis(2, at=seq(0,3000,by=300), lab=seq(0,3000,by=300),cex.axis=0.75, las=2,tck=-0.01)

#mtext("Cumulative Cases", side=2, line=3,cex=1)

mtext("C. Liberia", side=3, line=1,cex=0.8)

glev3=levels(liberia1$reg.id)

params=matrix(NA,length(glev3),15) # c(region,cexp,rexp,rexp_se,rstexp,c1poly,c2poly,mpoly,mpoly_se,rstpoly,klogi,clogi,rlogi,rlogi_se,rstlogi)

maxweek=1000

inc_all_liberia=rep(0,600)

v1=1:length(glev3)

for (i in v1){

sub_data=subset(liberia1,reg.id==glev3[i])

cases=sub_data$Numeric

case_data=cumsum(cases)

times=as.numeric(sub_data$dates22)

deltaT=7 ### time in between reports

while(length(which(diff(times)>deltaT))>0){

donde=which(diff(times)>deltaT)[1] # where in the vector of diff is it missing

cuando=times[donde] # what day

cuanto=diff(times)[donde] # how many days are missing

inser=cuanto/deltaT-1 # number of data points (weeks) to insert

new_cases=rep(NA,inser)

new_times=rep(NA,inser)

slope=(case_data[donde+1]-case_data[donde])/(cuanto)

interc=case_data[donde]

for (np in 1:inser){

new_cases[np]=deltaT*np*slope+interc

new_times[np]=cuando+deltaT*np

}

case_data=c(case_data[1:donde],new_cases,case_data[(donde+1):length(case_data)])

times=c(times[1:donde],new_times,times[(donde+1):length(times)])

} # end of while

case_data=cbind(case_data, times)

colnames(case_data)=c("cumcases","time")

case_data1=as.data.frame(case_data)

max_cases=tail(cumsum(cases),1)

min_time=min(as.numeric(sub_data$dates22))

case_data2=cbind(case_data1,case_data1$time-min_time+1 )

colnames(case_data2)=c("cumcases","time","time2")

case_data2=as.data.frame(case_data2)

# adding the flat tail at the "end" of the epidemic (up to July 2015) where no new cases were reported

inser2=(tmax0-max(case_data2$time))/deltaT-1

if(inser2>0){

case_data2_tail=as.data.frame(cbind(rep(max_cases,inser2+1),seq(max(case_data2$time)+deltaT,tmax0,deltaT),rep(NA,inser2+1)))

names(case_data2_tail)=c("cumcases","time","time2")

case_data2=rbind(case_data2,case_data2_tail)

}

points(case_data2$time,case_data2$cumcases, col="dimgray",bg=alpha(col_vec[i],0.5), pch=21,lwd=0.5, cex=0.4)

lines(case_data2$time,case_data2$cumcases, col=alpha(col_vec[i],0.4),lwd=2,lty=1)

legend(x=1,3200-(i-1)*130,legend=glev3[i],col=alpha(col_vec[i],0.7),lwd=2.5, cex=0.6,bty="n")

}

dev.off()

##################################################

# 3. Generate model fits of cumulative incidence

### Run models

### Guinea

for (we in c(6,10, 15) ){

mult.fig(1)

par(oma=c(1,1,1,1))

plot(unique(guinea1$dates22), rep(-1000,length(unique(guinea1$dates22))),xlab="", ylab="",ylim=c(0,130),xlim=c(0,350),cex.axis=0.9, cex=1.2,las=1)

mtext("Cumulative Cases", side=2, line=3,cex=1.2)

mtext("Days", side=1, line=2.5,cex=1.2)

mtext("Guinea", side=3, line=1,cex=1.5)

glev=levels(guinea1$reg.id)

params=matrix(NA,length(glev),15) # c(region,cexp,rexp,rexp_se,rstexp,c1poly,c2poly,mpoly,mpoly_se,rstpoly,klogi,clogi,rlogi,rlogi_se,rstlogi)

maxweek=we

v1=1:length(glev)

for (i in v1){

sub_data=subset(guinea1,reg.id==glev[i])

cases=sub_data$Numeric

#if(length(cases)>5){

case_data=cumsum(cases)

times=as.numeric(sub_data$dates22)

#plot(times,case_data)

deltaT=7 ### time in between reports

while(length(which(diff(times)>deltaT))>0){

donde=which(diff(times)>deltaT)[1] # where in the vector of diff is it missing

cuando=times[donde] # what day

cuanto=diff(times)[donde] # how many days are missing

inser=cuanto/deltaT-1 # number of data points (weeks) to insert

new_cases=rep(NA,inser)

new_times=rep(NA,inser)

slope=(case_data[donde+1]-case_data[donde])/(cuanto)

interc=case_data[donde]

for (np in 1:inser){

new_cases[np]=deltaT*np*slope+interc

new_times[np]=cuando+deltaT*np

}

case_data=c(case_data[1:donde],new_cases,case_data[(donde+1):length(case_data)])

times=c(times[1:donde],new_times,times[(donde+1):length(times)])

} # end of while

case_data=cbind(case_data, times)

colnames(case_data)=c("cumcases","time")

case_data1=as.data.frame(case_data)

max_cases=tail(cumsum(cases),1)

min_time=min(as.numeric(sub_data$dates22))

case_data2=cbind(case_data1,case_data1$time-min_time+1 )

colnames(case_data2)=c("cumcases","time","time2")

case_data2=as.data.frame(case_data2)

max_week1=min(maxweek,dim(case_data2)[1])

case_data2=case_data2[1:max_week1,] # take only the first two months of epidemic

#### EXPONENTIAL FIT

r0=.01 ## for the optimization algorithm

c0=2

cat(i,"\n")

try(expmod <- nls(cumcases ~ c*exp(r*time2),

data = case_data2,

start = list(c = c0, r = r0), control = list(maxiter = 1000)))

cexp=summary(expmod)$parameters[1]

rexp=summary(expmod)$parameters[2]

rexp_se=summary(expmod)$parameters[4]

#rss_exp=round(sum(resid(expmod)^2),2) # resisduals sums square

rse_exp=round(summary(expmod)$sigma,2) # residual standard error

params[i,1]=as.character(glev[i])

params[i,2]=cexp

params[i,3]=rexp

params[i,4]=rexp_se

params[i,5]=rse_exp

#### POLYNOMIAL FIT

st1 <- expand.grid(c1 = seq(-10, 10, len = 40),

c2 = seq(0, 100, len = 40), m = seq(0.0001, 2, len = 40))

polymod1 <- nls2(cumcases ~ c1 + c2*time2^m,

data = case_data2, start = st1, algorithm = "brute-force", control = list(maxiter = 1000))

cat("poly","\n")

try(

polymod1<-nls2(cumcases ~ c1 + c2*time2^m,

data = case_data2, start = coef(polymod1), control = list(maxiter = 1000))

)

c1=summary(polymod1)$parameters[1]

c2=summary(polymod1)$parameters[2]

m=summary(polymod1)$parameters[3]

m_se=summary(polymod1)$parameters[6]

#rss_poly=round(sum(resid(polymod1)^2),2)# resisduals sums square

rse_poly=round(summary(polymod1)$sigma,2) # residual standard error

params[i,6]=c1

params[i,7]=c2

params[i,8]=m

params[i,9]=m_se

params[i,10]=rse_poly

### LOGISTIC FIT

st2 <- expand.grid(k = seq(0, 1.5*max_cases, len = 40),

c = seq(0, 100, len = 40), r = seq(0.0001, 2, len = 40))

logimod1 <- nls2(cumcases ~ 1/(1/k + c*exp(-r*time2)),

data = case_data2, start = st2, algorithm = "brute-force", control = list(maxiter = 1000))

cat("logi","\n")

try(

logimod1<-nls2(cumcases ~ 1/(1/k + c*exp(-r*time2)),

data = case_data2, start = coef(logimod1), control = list(maxiter = 1000))

,silent=F)

k=summary(logimod1)$parameters[1]

c=summary(logimod1)$parameters[2]

r=summary(logimod1)$parameters[3]

r_se=summary(logimod1)$parameters[6]

#rss_logi=round(sum(resid(logimod1)^2),2) # resisduals sums square

rse_logi=round(summary(logimod1)$sigma,2) # residual standard error

params[i,11]=k

params[i,12]=c

params[i,13]=r

params[i,14]=r_se

params[i,15]=rse_logi

fit_fun=function(x){ cexp*exp(rexp*(x))}

xvs=1:max(case_data2$time2)

fit_line=fit_fun(xvs)

fit_fun_poly=function(x){ c1+c2*x^m}

fit_line_poly=fit_fun_poly(xvs)

fit_fun_logi=function(x){1/(1/k + c*exp(-r*(x)))}

fit_line_logi=fit_fun_logi(xvs)

points(case_data2$time,case_data2$cumcases, col=1,bg=alpha(col_vec[i],0.35), pch=21,lwd=0.7, cex=1.1)

lines(seq(min(case_data2$time),max(case_data2$time),by=1),fit_line, col=alpha(col_vec[i],1),lwd=3,lty=3)

lines(seq(min(case_data2$time),max(case_data2$time),by=1),fit_line_logi, col=alpha(col_vec[i],0.8),lwd=3)

legend(x=1,130-(i-1)*7,legend=glev[i],col=alpha(col_vec[i],0.7),lwd=3, cex=0.7,bty="n")

}

colnames(params)=c("region","cexp","rexp","rexp_se","rstexp","c1poly","c2poly","mpoly","mpoly_se","rstpoly","klogi","clogi","rlogi","rlogi_se","rstlogi")

write.csv(params, file = paste("table3_guinea_range_1to",maxweek,"_weeks",".csv",sep=""))

}

### Sierra

for (we in c(6, 10, 15) ){

mult.fig(1)

par(oma=c(1,1,1,1))

plot(unique(sierra1$dates22), rep(-1000,length(unique(sierra1$dates22))),xlab="", ylab="",ylim=c(0,570),xlim=c(0,350),cex.axis=0.9, cex=1.2,las=1)

mtext("Cumulative Cases", side=2, line=3,cex=1.2)

mtext("Days", side=1, line=2.5,cex=1.2)

mtext("Sierra Leone", side=3, line=1,cex=1.5)

glev=levels(sierra1$reg.id)

params=matrix(NA,length(glev),15) # c(region,cexp,rexp,rexp_se,rstexp,c1poly,c2poly,mpoly,mpoly_se,rstpoly,klogi,clogi,rlogi,rlogi_se,rstlogi)

maxweek=we

v1=1:length(glev)

for (i in v1){

sub_data=subset(sierra1,reg.id==glev[i])

cases=sub_data$Numeric

case_data=cumsum(cases)

times=as.numeric(sub_data$dates22)

deltaT=7 ### time in between reports

while(length(which(diff(times)>deltaT))>0){

donde=which(diff(times)>deltaT)[1] # where in the vector of diff is it missing

cuando=times[donde] # what day

cuanto=diff(times)[donde] # how many days are missing

inser=cuanto/deltaT-1 # number of data points (weeks) to insert

new_cases=rep(NA,inser)

new_times=rep(NA,inser)

slope=(case_data[donde+1]-case_data[donde])/(cuanto)

interc=case_data[donde]

for (np in 1:inser){

new_cases[np]=deltaT*np*slope+interc

new_times[np]=cuando+deltaT*np

}

case_data=c(case_data[1:donde],new_cases,case_data[(donde+1):length(case_data)])

times=c(times[1:donde],new_times,times[(donde+1):length(times)])

} # end of while

case_data=cbind(case_data, times)

colnames(case_data)=c("cumcases","time")

case_data1=as.data.frame(case_data)

max_cases=tail(cumsum(cases),1)

min_time=min(as.numeric(sub_data$dates22))

case_data2=cbind(case_data1,case_data1$time-min_time+1 )

colnames(case_data2)=c("cumcases","time","time2")

case_data2=as.data.frame(case_data2)

max_week1=min(maxweek,dim(case_data2)[1])

case_data2=case_data2[1:max_week1,]

#### EXPONENTIAL FIT

r0=.01 ## for the optimization algorithm

c0=2

cat(i,"\n")

try(expmod <- nls(cumcases ~ c*exp(r*time2),

data = case_data2,

start = list(c = c0, r = r0), control = list(maxiter = 1000)))

cexp=summary(expmod)$parameters[1]

rexp=summary(expmod)$parameters[2]

rexp_se=summary(expmod)$parameters[4]

#rss_exp=round(sum(resid(expmod)^2),2) # resisduals sums square

rse_exp=round(summary(expmod)$sigma,2) # residual standard error

params[i,1]=as.character(glev[i])

params[i,2]=cexp

params[i,3]=rexp

params[i,4]=rexp_se

params[i,5]=rse_exp

#### POLYNOMIAL FIT

st1 <- expand.grid(c1 = seq(-10, 10, len = 40),

c2 = seq(0, 100, len = 40), m = seq(0.0001, 2, len = 40))

polymod1 <- nls2(cumcases ~ c1 + c2*time2^m,

data = case_data2, start = st1, algorithm = "brute-force", control = list(maxiter = 1000))

cat("poly","\n")

try(

polymod1<-nls2(cumcases ~ c1 + c2*time2^m,

data = case_data2, start = coef(polymod1), control = list(maxiter = 1000))

)

c1=summary(polymod1)$parameters[1]

c2=summary(polymod1)$parameters[2]

m=summary(polymod1)$parameters[3]

m_se=summary(polymod1)$parameters[6]

#rss_poly=round(sum(resid(polymod1)^2),2)# resisduals sums square

rse_poly=round(summary(polymod1)$sigma,2) # residual standard error

params[i,6]=c1

params[i,7]=c2

params[i,8]=m

params[i,9]=m_se

params[i,10]=rse_poly

### LOGISTIC FIT

st2 <- expand.grid(k = seq(0, 1.5*max_cases, len = 40),

c = seq(0, 10, len = 40), r = seq(0.0001, 2, len = 40))

#st2 <- data.frame(k = c(0, ), c = c(-100, 100), r = c(0.0001, 2))

logimod1 <- nls2(cumcases ~ 1/(1/k + c*exp(-r*time2)),

data = case_data2, start = st2, algorithm = "brute-force", control = list(maxiter = 1000))

cat("logi","\n")

try(

logimod1<-nls2(cumcases ~ 1/(1/k + c*exp(-r*time2)),

data = case_data2, start = coef(logimod1), control = list(maxiter = 1000))

,silent=F)

k=summary(logimod1)$parameters[1]

c=summary(logimod1)$parameters[2]

r=summary(logimod1)$parameters[3]

r_se=summary(logimod1)$parameters[6]

#rss_logi=round(sum(resid(logimod1)^2),2) # resisduals sums square

rse_logi=round(summary(logimod1)$sigma,2) # residual standard error

params[i,11]=k

params[i,12]=c

params[i,13]=r

params[i,14]=r_se

params[i,15]=rse_logi

fit_fun=function(x){ cexp*exp(rexp*(x))}

xvs=1:max(case_data2$time2)

fit_line=fit_fun(xvs)

fit_fun_poly=function(x){ c1+c2*x^m}

fit_line_poly=fit_fun_poly(xvs)

fit_fun_logi=function(x){1/(1/k + c*exp(-r*(x)))}

fit_line_logi=fit_fun_logi(xvs)

points(case_data2$time,case_data2$cumcases, col=1,bg=alpha(col_vec[i],0.35), pch=21,lwd=0.7, cex=1.1)

lines(seq(min(case_data2$time),max(case_data2$time),by=1),fit_line, col=alpha(col_vec[i],1),lwd=3,lty=3)

#lines(seq(min(case_data2$time),max(case_data2$time),by=1),fit_line_poly, col=alpha(col_vec[i],1),lwd=2)

lines(seq(min(case_data2$time),max(case_data2$time),by=1),fit_line_logi, col=alpha(col_vec[i],0.8),lwd=3)

legend(x=1,570-(i-1)*22,legend=glev[i],col=alpha(col_vec[i],0.7),lwd=3, cex=0.7,bty="n")

}

colnames(params)=c("region","cexp","rexp","rexp_se","rstexp","c1poly","c2poly","mpoly","mpoly_se","rstpoly","klogi","clogi","rlogi","rlogi_se","rstlogi")

write.csv(params, file = paste("table3_sierra_range_1to",maxweek,"_weeks",".csv",sep=""))

}

### Liberia

for (we in c(6, 10, 15) ){

mult.fig(1)

par(oma=c(1,1,1,1))

plot(unique(liberia1$dates22), rep(-1000,length(unique(liberia1$dates22))),xlab="", ylab="",xlim=c(0,350),ylim=c(0,320),cex.axis=0.9, cex=1.2,las=1)

mtext("Cumulative Cases", side=2, line=3,cex=1.2)

mtext("Days", side=1, line=2.5,cex=1.2)

mtext("Liberia", side=3, line=1,cex=1.5)

glev=levels(liberia1$reg.id)

params=matrix(NA,length(glev),15) # c(region,cexp,rexp,rexp_se,rstexp,c1poly,c2poly,mpoly,mpoly_se,rstpoly,klogi,clogi,rlogi,rlogi_se,rstlogi)

maxweek=we

v1=1:length(glev)

for (i in v1){

sub_data=subset(liberia1,reg.id==glev[i])

cases=sub_data$Numeric

case_data=cumsum(cases)

times=as.numeric(sub_data$dates22)

deltaT=7 ### time in between reports

while(length(which(diff(times)>deltaT))>0){

donde=which(diff(times)>deltaT)[1] # where in the vector of diff is it missing

cuando=times[donde] # what day

cuanto=diff(times)[donde] # how many days are missing

inser=cuanto/deltaT-1 # number of data points (weeks) to insert

new_cases=rep(NA,inser)

new_times=rep(NA,inser)

slope=(case_data[donde+1]-case_data[donde])/(cuanto)

interc=case_data[donde]

for (np in 1:inser){

new_cases[np]=deltaT*np*slope+interc

new_times[np]=cuando+deltaT*np

}

case_data=c(case_data[1:donde],new_cases,case_data[(donde+1):length(case_data)])

times=c(times[1:donde],new_times,times[(donde+1):length(times)])

} # end of while

case_data=cbind(case_data, times)

colnames(case_data)=c("cumcases","time")

case_data1=as.data.frame(case_data)

max_cases=tail(cumsum(cases),1)

min_time=min(as.numeric(sub_data$dates22))

case_data2=cbind(case_data1,case_data1$time-min_time+1 )

colnames(case_data2)=c("cumcases","time","time2")

case_data2=as.data.frame(case_data2)

max_week1=min(maxweek,dim(case_data2)[1])

case_data2=case_data2[1:max_week1,] # take only the first two months of epidemic

#### EXPONENTIAL FIT

r0=.01 ## for the optimization algorithm

c0=2

cat(i,"\n")

try(expmod <- nls(cumcases ~ c*exp(r*time2),

data = case_data2,

start = list(c = c0, r = r0), control = list(maxiter = 1000)))

cexp=summary(expmod)$parameters[1]

rexp=summary(expmod)$parameters[2]

rexp_se=summary(expmod)$parameters[4]

#rss_exp=round(sum(resid(expmod)^2),2) # resisduals sums square

rse_exp=round(summary(expmod)$sigma,2) # residual standard error

params[i,1]=as.character(glev[i])

params[i,2]=cexp

params[i,3]=rexp

params[i,4]=rexp_se

params[i,5]=rse_exp

#### POLYNOMIAL FIT

st1 <- expand.grid(c1 = seq(-10, 10, len = 40),

c2 = seq(0, 100, len = 40), m = seq(0.0001, 2, len = 40))

polymod1 <- nls2(cumcases ~ c1 + c2*time2^m,

data = case_data2, start = st1, algorithm = "brute-force", control = list(maxiter = 1000))

cat("poly","\n")

try(

polymod1<-nls2(cumcases ~ c1 + c2*time2^m,

data = case_data2, start = coef(polymod1), control = list(maxiter = 1000))

)

c1=summary(polymod1)$parameters[1]

c2=summary(polymod1)$parameters[2]

m=summary(polymod1)$parameters[3]

m_se=summary(polymod1)$parameters[6]

#rss_poly=round(sum(resid(polymod1)^2),2)# resisduals sums square

rse_poly=round(summary(polymod1)$sigma,2) # residual standard error

params[i,6]=c1

params[i,7]=c2

params[i,8]=m

params[i,9]=m_se

params[i,10]=rse_poly

### LOGISTIC FIT

st2 <- expand.grid(k = seq(0, 1.5*max_cases, len = 40),

c = seq(0, 100, len = 40), r = seq(0.0001, 2, len = 40))

logimod1 <- nls2(cumcases ~ 1/(1/k + c*exp(-r*time2)),

data = case_data2, start = st2, algorithm = "brute-force", control = list(maxiter = 1000))

cat("logi","\n")

try(

logimod1<-nls2(cumcases ~ 1/(1/k + c*exp(-r*time2)),

data = case_data2, start = coef(logimod1), control = list(maxiter = 1000))

,silent=F)

k=summary(logimod1)$parameters[1]

c=summary(logimod1)$parameters[2]

r=summary(logimod1)$parameters[3]

r_se=summary(logimod1)$parameters[6]

#rss_logi=round(sum(resid(logimod1)^2),2) # resisduals sums square

rse_logi=round(summary(logimod1)$sigma,2) # residual standard error

params[i,11]=k

params[i,12]=c

params[i,13]=r

params[i,14]=r_se

params[i,15]=rse_logi

fit_fun=function(x){ cexp*exp(rexp*(x))}

xvs=1:max(case_data2$time2)

fit_line=fit_fun(xvs)

fit_fun_poly=function(x){ c1+c2*x^m}

fit_line_poly=fit_fun_poly(xvs)

fit_fun_logi=function(x){1/(1/k + c*exp(-r*(x)))}

fit_line_logi=fit_fun_logi(xvs)

points(case_data2$time,case_data2$cumcases, col=1,bg=alpha(col_vec[i],0.35), pch=21,lwd=0.7, cex=1.1)

#lines(seq(min(case_data2$time),max(case_data2$time),by=1),fit_line, col=alpha(col_vec[i],1),lwd=3,lty=3)

lines(seq(min(case_data2$time),max(case_data2$time),by=1),fit_line_poly, col=alpha(col_vec[i],1),lwd=2)

#lines(seq(min(case_data2$time),max(case_data2$time),by=1),fit_line_logi, col=alpha(col_vec[i],0.8),lwd=3)

legend(x=1,320-(i-1)*15,legend=glev[i],col=alpha(col_vec[i],0.7),lwd=3, cex=0.7,bty="n")

}

colnames(params)=c("region","cexp","rexp","rexp_se","rstexp","c1poly","c2poly","mpoly","mpoly_se","rstpoly","klogi","clogi","rlogi","rlogi_se","rstlogi")

write.csv(params, file = paste("table3_liberia_range_1to",maxweek,"_weeks",".csv",sep=""))

}

######################################################

# 4. Plot cumulative curves for each type of model

### reading tables with fits results

guinea_tab6=read.csv("table3_guinea_range_1to6_weeks.csv")

guinea_tab10=read.csv("table3_guinea_range_1to10_weeks.csv")

guinea_tab15=read.csv("table3_guinea_range_1to15_weeks.csv")

sierra_tab6=read.csv("table3_sierra_range_1to6_weeks.csv")

sierra_tab10=read.csv("table3_sierra_range_1to10_weeks.csv")

sierra_tab15=read.csv("table3_sierra_range_1to15_weeks.csv")

liberia_tab6=read.csv("table3_liberia_range_1to6_weeks.csv")

liberia_tab10=read.csv("table3_liberia_range_1to10_weeks.csv")

liberia_tab15=read.csv("table3_liberia_range_1to15_weeks.csv")

### Run plots

# cairo_pdf("Fig2_v5.eps",width=7.5, height=2.5,bg="transparent")

tiff("Fig2_v5.tiff", units="in", width=7.5, height=2.5, pointsize = 12,res=350)

mult.fig(mfrow=c(1,3))

par(oma=c(1,1,1,1))

par(mai=c(.3,.5,.3,0))

# Guinea

#for (we in c(6,10, 15) ){

for (we in 6 ){

#ymax=125

ymax=50

plot(unique(guinea1$dates22), rep(-1000,length(unique(guinea1$dates22))),xlab="", ylab="",ylim=c(0,ymax),xlim=c(0,300),cex.axis=0.9, cex=1.2,las=1,tck=-0.01)

mtext("Cumulative Cases", side=2, line=3,cex=0.6)

mtext("Days", side=1, line=2,cex=0.6)

mtext("A. Guinea", side=3, line=1,cex=0.8)

glev=levels(guinea1$reg.id)

maxweek=we

v1=1:length(glev)

for (i in v1){

sub_data=subset(guinea1,reg.id==glev[i])

cases=sub_data$Numeric

case_data=cumsum(cases)

times=as.numeric(sub_data$dates22)

deltaT=7 ### time in between reports

while(length(which(diff(times)>deltaT))>0){

donde=which(diff(times)>deltaT)[1] # where in the vector of diff is it missing

cuando=times[donde] # what day

cuanto=diff(times)[donde] # how many days are missing

inser=cuanto/deltaT-1 # number of data points (weeks) to insert

new_cases=rep(NA,inser)

new_times=rep(NA,inser)

slope=(case_data[donde+1]-case_data[donde])/(cuanto)

interc=case_data[donde]

for (np in 1:inser){

new_cases[np]=deltaT*np*slope+interc

new_times[np]=cuando+deltaT*np

}

case_data=c(case_data[1:donde],new_cases,case_data[(donde+1):length(case_data)])

times=c(times[1:donde],new_times,times[(donde+1):length(times)])

} # end of while

case_data=cbind(case_data, times)

colnames(case_data)=c("cumcases","time")

case_data1=as.data.frame(case_data)

max_cases=tail(cumsum(cases),1)

min_time=min(as.numeric(sub_data$dates22))

case_data2=cbind(case_data1,case_data1$time-min_time+1 )

colnames(case_data2)=c("cumcases","time","time2")

case_data2=as.data.frame(case_data2)

max_week1=min(maxweek,dim(case_data2)[1])

case_data2=case_data2[1:max_week1,] # take only the first two months of epidemic

if (maxweek==6) guinea_tab=guinea_tab6

if (maxweek==10) guinea_tab=guinea_tab10

if (maxweek==15) guinea_tab=guinea_tab15

#### EXPONENTIAL FIT

cexp=guinea_tab[i,3]

rexp=guinea_tab[i,4]

#### POLYNOMIAL FIT

c1=guinea_tab[i,7]

c2=guinea_tab[i,8]

m=guinea_tab[i,9]

### LOGISTIC FIT

k=guinea_tab[i,12]

c=guinea_tab[i,13]

r=guinea_tab[i,14]

fit_fun=function(x){ cexp*exp(rexp*(x))}

xvs=1:max(case_data2$time2)

fit_line=fit_fun(xvs)

fit_fun_poly=function(x){ c1+c2*x^m}

fit_line_poly=fit_fun_poly(xvs)

fit_fun_logi=function(x){1/(1/k + c*exp(-r*(x)))}

fit_line_logi=fit_fun_logi(xvs)

points(case_data2$time,case_data2$cumcases, col="dimgray",bg=alpha(col_vec[i],0.35), pch=21,lwd=0.3, cex=0.8)

#lines(seq(min(case_data2$time),max(case_data2$time),by=1),fit_line, col=alpha(col_vec[i],1),lwd=3,lty=3)

lines(seq(min(case_data2$time),max(case_data2$time),by=1),fit_line_poly, col=alpha(col_vec[i],1),lwd=2)

#lines(seq(min(case_data2$time),max(case_data2$time),by=1),fit_line_logi, col=alpha(col_vec[i],0.8),lwd=3)

legend(x=1,ymax-(i-1)*(ymax/20)+3,legend=glev[i],col=alpha(col_vec[i],0.9),lwd=2.5, cex=0.6,bty="n")

}

}

### Sierra Leone

#for (we in c(6,10, 15) ){

for (we in 6 ){

#ymax=600

ymax=250

plot(unique(sierra1$dates22), rep(-1000,length(unique(sierra1$dates22))),xlab="", ylab="",ylim=c(0,ymax),xlim=c(0,300),cex.axis=0.9, cex=1.2,las=1,tck=-0.01)

#mtext("Cumulative Cases", side=2, line=3,cex=1.2)

mtext("Days", side=1, line=2,cex=0.6)

mtext("B. Sierra Leone", side=3, line=1,cex=0.8)

glev=levels(sierra1$reg.id)

glev2=glev

glev2[c(13,14)]=c("WEST. AREA RURAL","WEST. AREA URBAN") # for legend

maxweek=we

v1=1:length(glev)

for (i in v1){

sub_data=subset(sierra1,reg.id==glev[i])

cases=sub_data$Numeric

case_data=cumsum(cases)

times=as.numeric(sub_data$dates22)

deltaT=7 ### time in between reports

while(length(which(diff(times)>deltaT))>0){

donde=which(diff(times)>deltaT)[1] # where in the vector of diff is it missing

cuando=times[donde] # what day

cuanto=diff(times)[donde] # how many days are missing

inser=cuanto/deltaT-1 # number of data points (weeks) to insert

new_cases=rep(NA,inser)

new_times=rep(NA,inser)

slope=(case_data[donde+1]-case_data[donde])/(cuanto)

interc=case_data[donde]

for (np in 1:inser){

new_cases[np]=deltaT*np*slope+interc

new_times[np]=cuando+deltaT*np

}

case_data=c(case_data[1:donde],new_cases,case_data[(donde+1):length(case_data)])

times=c(times[1:donde],new_times,times[(donde+1):length(times)])

} # end of while

case_data=cbind(case_data, times)

colnames(case_data)=c("cumcases","time")

case_data1=as.data.frame(case_data)

max_cases=tail(cumsum(cases),1)

min_time=min(as.numeric(sub_data$dates22))

case_data2=cbind(case_data1,case_data1$time-min_time+1 )

colnames(case_data2)=c("cumcases","time","time2")

case_data2=as.data.frame(case_data2)

max_week1=min(maxweek,dim(case_data2)[1])

case_data2=case_data2[1:max_week1,] # take only the first two months of epidemic

if (maxweek==6) sierra_tab=sierra_tab6

if (maxweek==10) sierra_tab=sierra_tab10

if (maxweek==15) sierra_tab=sierra_tab15

#### EXPONENTIAL FIT

cexp=sierra_tab[i,3]

rexp=sierra_tab[i,4]

#### POLYNOMIAL FIT

c1=sierra_tab[i,7]

c2=sierra_tab[i,8]

m=sierra_tab[i,9]

### LOGISTIC FIT

k=sierra_tab[i,12]

c=sierra_tab[i,13]

r=sierra_tab[i,14]

fit_fun=function(x){ cexp*exp(rexp*(x))}

xvs=1:max(case_data2$time2)

fit_line=fit_fun(xvs)

fit_fun_poly=function(x){ c1+c2*x^m}

fit_line_poly=fit_fun_poly(xvs)

fit_fun_logi=function(x){1/(1/k + c*exp(-r*(x)))}

fit_line_logi=fit_fun_logi(xvs)

points(case_data2$time,case_data2$cumcases, col="dimgray",bg=alpha(col_vec[i],0.35), pch=21,lwd=0.4, cex=0.8)

#lines(seq(min(case_data2$time),max(case_data2$time),by=1),fit_line, col=alpha(col_vec[i],1),lwd=3,lty=3)

lines(seq(min(case_data2$time),max(case_data2$time),by=1),fit_line_poly, col=alpha(col_vec[i],1),lwd=2)

#lines(seq(min(case_data2$time),max(case_data2$time),by=1),fit_line_logi, col=alpha(col_vec[i],0.8),lwd=3)

legend(x=1,ymax-(i-1)*(ymax/20)+15,legend=glev2[i],col=alpha(col_vec[i],0.9),lwd=2.5, cex=0.6,bty="n")

}

}

### Liberia

#for (we in c(6,10, 15) ){

for (we in 6 ){

ymax=50

plot(unique(liberia1$dates22), rep(-1000,length(unique(liberia1$dates22))),xlab="", ylab="",xlim=c(0,300),ylim=c(0,ymax),cex.axis=0.9, cex=1.2,las=1,tck=-0.01)

#mtext("Cumulative Cases", side=2, line=3,cex=1.2)

mtext("Days", side=1, line=2,cex=0.6)

mtext("C. Liberia", side=3, line=1,cex=0.8)

glev=levels(liberia1$reg.id)

maxweek=we

v1=1:length(glev)

for (i in v1){

sub_data=subset(liberia1,reg.id==glev[i])

cases=sub_data$Numeric

#if(length(cases)>5){

case_data=cumsum(cases)

times=as.numeric(sub_data$dates22)

#plot(times,case_data)

deltaT=7 ### time in between reports

while(length(which(diff(times)>deltaT))>0){

donde=which(diff(times)>deltaT)[1] # where in the vector of diff is it missing

cuando=times[donde] # what day

cuanto=diff(times)[donde] # how many days are missing

inser=cuanto/deltaT-1 # number of data points (weeks) to insert

new_cases=rep(NA,inser)

new_times=rep(NA,inser)

slope=(case_data[donde+1]-case_data[donde])/(cuanto)

interc=case_data[donde]

for (np in 1:inser){

new_cases[np]=deltaT*np*slope+interc

new_times[np]=cuando+deltaT*np

}

case_data=c(case_data[1:donde],new_cases,case_data[(donde+1):length(case_data)])

times=c(times[1:donde],new_times,times[(donde+1):length(times)])

} # end of while

case_data=cbind(case_data, times)

colnames(case_data)=c("cumcases","time")

case_data1=as.data.frame(case_data)

max_cases=tail(cumsum(cases),1)

min_time=min(as.numeric(sub_data$dates22))

case_data2=cbind(case_data1,case_data1$time-min_time+1 )

colnames(case_data2)=c("cumcases","time","time2")

case_data2=as.data.frame(case_data2)

max_week1=min(maxweek,dim(case_data2)[1])

case_data2=case_data2[1:max_week1,] # take only the first two months of epidemic

if (maxweek==6) liberia_tab=liberia_tab6

if (maxweek==10) liberia_tab=liberia_tab10

if (maxweek==15) liberia_tab=liberia_tab15

#### EXPONENTIAL FIT

cexp=liberia_tab[i,3]

rexp=liberia_tab[i,4]

#### POLYNOMIAL FIT

c1=liberia_tab[i,7]

c2=liberia_tab[i,8]

m=liberia_tab[i,9]

### LOGISTIC FIT

k=liberia_tab[i,12]

c=liberia_tab[i,13]

r=liberia_tab[i,14]

fit_fun=function(x){ cexp*exp(rexp*(x))}

xvs=1:max(case_data2$time2)

fit_line=fit_fun(xvs)

fit_fun_poly=function(x){ c1+c2*x^m}

fit_line_poly=fit_fun_poly(xvs)

fit_fun_logi=function(x){1/(1/k + c*exp(-r*(x)))}

fit_line_logi=fit_fun_logi(xvs)

points(case_data2$time,case_data2$cumcases, col="dimgray",bg=alpha(col_vec[i],0.35), pch=21,lwd=0.4, cex=0.8)

#lines(seq(min(case_data2$time),max(case_data2$time),by=1),fit_line, col=alpha(col_vec[i],1),lwd=3,lty=3)

lines(seq(min(case_data2$time),max(case_data2$time),by=1),fit_line_poly, col=alpha(col_vec[i],1),lwd=2)

#lines(seq(min(case_data2$time),max(case_data2$time),by=1),fit_line_logi, col=alpha(col_vec[i],0.8),lwd=3)

legend(x=1,ymax-(i-1)*(ymax/20)+3,legend=glev[i],col=alpha(col_vec[i],0.9),lwd=2.5, cex=0.6,bty="n")

}

}

dev.off()

Section 4. Tidy the DHS data and merge it with wealth and population data, and with Ebola curve summary statistics. Code written in R version 3.2 and Stata version 13

##Code for Generating the dataset for final analysis

#1. First, indicator variables are generated from DHS data.

#2. Second, newly generated covariate data is weighted in STATA ***(see code at end of section)

#3. Third, weighted covariate data is merged with the data of estimated rates from various weeks

library("foreign")

x<-read.dta(file="MergedDataset.dta", convert.dates = TRUE, convert.factors = TRUE,

missing.type = FALSE, convert.underscore = FALSE, warn.missing.labels = TRUE)

x<-x[which(x$country!="Mali" & x$country!="Senegal"& x$country!="CoteDIvoire" ),]

################

#create indicator variables

x$radio_own_new<-(as.numeric(x$radio_own)-1)

x$female_new<-(as.numeric(x$female)-1)

x$urban_new<-(as.numeric(x$urban)-1)

x$bicycle_new<-(as.numeric(x$urban)-1)

x$motorbike_new<-(as.numeric(x$urban)-1)

x$car_new<-(as.numeric(x$urban)-1)

x$religion_new_1<-as.numeric(x$religion)

x$religion_new_2<-as.numeric(x$religion)

x$religion_new_3<-as.numeric(x$religion)

x$religion_new_1[which(as.numeric(x$religion)!=10)]<-0

x$religion_new_1[which(as.numeric(x$religion)==10)]<-1

x$religion_new_2[which(as.numeric(x$religion)!=5)]<-0

x$religion_new_2[which(as.numeric(x$religion)==5)]<-1

x$religion_new_3[which(as.numeric(x$religion)==5 | as.numeric(x$religion)==10)]<-0

x$religion_new_3[which(as.numeric(x$religion)!=5 & as.numeric(x$religion)!=10)]<-1

x$religion_new_1<-as.numeric(x$religion)

x$religion_new_2<-as.numeric(x$religion)

x$religion_new_3<-as.numeric(x$religion)

x$religion_new_1[which(as.numeric(x$religion)!=10)]<-0

x$religion_new_1[which(as.numeric(x$religion)==10)]<-1

x$religion_new_2[which(as.numeric(x$religion)!=5)]<-0

x$religion_new_2[which(as.numeric(x$religion)==5)]<-1

x$religion_new_3[which(as.numeric(x$religion)==5 | as.numeric(x$religion)==10)]<-0

x$religion_new_3[which(as.numeric(x$religion)!=5 & as.numeric(x$religion)!=10)]<-1

x$toilet_new<-as.numeric(x$toilet)

x$toilet_new[which(as.numeric(x$toilet)!=10)]<-0

x$toilet_new[which(as.numeric(x$toilet)==10)]<-1

x$wealth_new_1<-as.numeric(x$wealth_q)

x$wealth_new_2<-as.numeric(x$wealth_q)

x$wealth_new_3<-as.numeric(x$wealth_q)

x$wealth_new_4<-as.numeric(x$wealth_q)

x$wealth_new_5<-as.numeric(x$wealth_q)

x$wealth_new_1[which(as.numeric(x$wealth_q)!=1)]<-0

x$wealth_new_2[which(as.numeric(x$wealth_q)!=2)]<-0

x$wealth_new_2[which(as.numeric(x$wealth_q)==2)]<-1

x$wealth_new_3[which(as.numeric(x$wealth_q)!=3)]<-0

x$wealth_new_3[which(as.numeric(x$wealth_q)==3)]<-1

x$wealth_new_4[which(as.numeric(x$wealth_q)!=4)]<-0

x$wealth_new_4[which(as.numeric(x$wealth_q)==4)]<-1

x$wealth_new_5[which(as.numeric(x$wealth_q)!=5)]<-0

x$wealth_new_5[which(as.numeric(x$wealth_q)==5)]<-1

x$educat_new_1<-as.numeric(x$educat)

x$educat_new_2<-as.numeric(x$educat)

x$educat_new_3<-as.numeric(x$educat)

x$educat_new_4<-as.numeric(x$educat)

x$educat_new_1[which(as.numeric(x$educat)!=1)]<-0

x$educat_new_2[which(as.numeric(x$educat)!=2)]<-0

x$educat_new_2[which(as.numeric(x$educat)==2)]<-1

x$educat_new_3[which(as.numeric(x$educat)!=3)]<-0

x$educat_new_3[which(as.numeric(x$educat)==3)]<-1

x$educat_new_4[which(as.numeric(x$educat)!=4)]<-0

x$educat_new_4[which(as.numeric(x$educat)==4)]<-1

x$radio_listen_new_1<-as.numeric(x$radio_listen)

x$radio_listen_new_2<-as.numeric(x$radio_listen)

x$radio_listen_new_3<-as.numeric(x$radio_listen)

x$radio_listen_new_1[which(as.numeric(x$radio_listen)!=1)]<-0

x$radio_listen_new_2[which(as.numeric(x$radio_listen)!=2)]<-0

x$radio_listen_new_2[which(as.numeric(x$radio_listen)==2)]<-1

x$radio_listen_new_3[which(as.numeric(x$radio_listen)!=3)]<-0

x$radio_listen_new_3[which(as.numeric(x$radio_listen)==3)]<-1

new_data_stratum<-data.frame(x$stratum,as.numeric(x$toilet_new),as.numeric(x$radio_own_new),x$age,x$female_new,x$urban_new,x$bicycle_new,x$motorbike_new, x$car_new,x$wealth_new_1,x$wealth_new_2,x$wealth_new_3,x$wealth_new_4,x$wealth_new_5,

as.numeric(x$educat_new_1),as.numeric(x$educat_new_2),as.numeric(x$educat_new_3),as.numeric(x$educat_new_4), x$radio_listen_new_1,x$radio_listen_new_2,x$radio_listen_new_3, as.numeric(x$religion_new_1),as.numeric(x$religion_new_2),as.numeric(x$religion_new_3),x$wgt_indiv,x$region,x$wgt_sex_specifi)

colnames(new_data_stratum)<-c("stratum","toilet","radio_own","age","female","urban","bicycle","motorbike", "car", "poorest","poorer","middle",

"richer", "richest", "no_edu", "primary_ed","secondary_ed", "higher_ed","no_radio_listen",

"at_least_once_week_radio_listen","always_radio_listen","muslim","christian","other_religion","wgt_indiv","region","wgt_sex_specifi")

write.csv(new_data_stratum,"data_dhs_for_aggregation_revision_august18.csv")

###Input above data into STATA to perform the weighting

##****##Follow the do file at end of this section

###Output file: weighted_merged_data_aug18.csv

## NEW AGGREGATED DATA

## Read in DHS aggregated data

ebola.dhs.aggregate<-read.csv("weighted_merged_data_aug18.csv")

xbis<-as.matrix(ebola.dhs.aggregate)

xbis[2,1]<-"Boke"

xbis[21,1]<-"Labe"

xbis[28,1]<-"N'Zerekore"

new_data_region<-as.data.frame(xbis)

new_data_region$reg.id<-toupper(new_data_region$region)

## Read in Density Data:

ebola.n<-read.csv(file="population_census.csv",head=TRUE,sep=",")

dhs_data<-merge(new_data_region,ebola.n,by="reg.id")

#### DHS Data + Rates

#Combine Rates Across Countries & Time Span

guinea1to15<-read.csv(file="table3_guinea_range_1to15_weeks.csv",header=TRUE)

guinea1to15$uptoweek<-15

guinea1to10<-read.csv(file="table3_guinea_range_1to10_weeks.csv",header=TRUE)

guinea1to10$uptoweek<-10

guinea1to6<-read.csv(file="table3_guinea_range_1to6_weeks.csv",header=TRUE)

guinea1to6$uptoweek<-6

liberia1to15<-read.csv(file="table3_liberia_range_1to15_weeks.csv",header=TRUE)

liberia1to15$uptoweek<-15

liberia1to10<-read.csv(file="table3_liberia_range_1to10_weeks.csv",header=TRUE)

liberia1to10$uptoweek<-10

liberia1to6<-read.csv(file="table3_liberia_range_1to6_weeks.csv",header=TRUE)

liberia1to6$uptoweek<-6

sierra1to15<-read.csv(file="table3_sierra_range_1to15_weeks.csv",header=TRUE)

sierra1to15$uptoweek<-15

sierra1to10<-read.csv(file="table3_sierra_range_1to10_weeks.csv",header=TRUE)

sierra1to10$uptoweek<-10

sierra1to6<-read.csv(file="table3_sierra_range_1to6_weeks.csv",header=TRUE)

sierra1to6$uptoweek<-6

rate_est<-rbind(guinea1to15,guinea1to6,liberia1to15,liberia1to6,sierra1to15,sierra1to6)

rate_est$reg.id<-as.character(rate_est$region)

rate_est$reg.id[which(rate_est$reg.id=="RIVERCESS")]<-"RIVER CESS"

rate_est$reg.id[which(rate_est$reg.id=="WESTERN AREA RURAL")]<-"WESTERN RURAL"

rate_est$reg.id[which(rate_est$reg.id=="WESTERN AREA URBAN")]<-"WESTERN URBAN"

#bonthe and maryland missing because of low counts

#Combine DHS, Covariates, Rates for Estimates

merged<-merge(dhs_data,rate_est,by="reg.id")

write.csv(merged,file="merged_cov_rates_revision_august18.csv")

## Merge numeric start outbreak

sierra.t<-read.csv("sierra.aggregated_july.csv")

liberia.t<-read.csv("liberia.aggregated_july.csv")

guinea.t<-read.csv("guinea.aggregated_july.csv")

t.data<-as.data.frame(rbind(sierra.t, liberia.t, guinea.t))

t.data$reg.id<-as.character(t.data$reg.id)

t.data$reg.id[which(t.data$reg.id=="RIVERCESS")]<-"RIVER CESS"

t.data$reg.id[which(t.data$reg.id=="WESTERN AREA RURAL")]<-"WESTERN RURAL"

t.data$reg.id[which(t.data$reg.id=="WESTERN AREA URBAN")]<-"WESTERN URBAN"

## Merge iwi, edu, start outbreak

sierra.iwi<-read.csv("sierra.iwi.csv")[,-1] #Delete index row

liberia.iwi<-read.csv("liberia.iwi.csv")[,-1] #Delete index row

guinea.iwi<-read.csv("guinea.iwi.csv")[,-1] #Delete index row

iwi.data<-as.data.frame(rbind(sierra.iwi, liberia.iwi, guinea.iwi))

iwi.data$reg.id<-as.character(iwi.data$reg.id)

iwi.data$reg.id[which(iwi.data$reg.id=="RIVERCESS")]<-"RIVER CESS"

iwi.data$reg.id[which(iwi.data$reg.id=="WESTERN AREA RURAL")]<-"WESTERN RURAL"

iwi.data$reg.id[which(iwi.data$reg.id=="WESTERN AREA URBAN")]<-"WESTERN URBAN"

merged_t<-merge(iwi.data,merged,by="reg.id")

merged_t<-merge(t.data,merged_t,by="reg.id")

write.csv(merged_t, file="merged_cov_rates_updated_time_series_revision_august_final.csv")

###############################

# DO file to be used in middle of R code above.

* Import R Data First

import delimited "data_dhs_for_aggregation_revision_august18.csv", encoding(ISO-8859-1)

* Change to Numeric Variable

destring , replace ignore(NA)

* Apply the Weighting function after

collapse (mean) toilet age female urban radio_own bicycle car motorbike poorest poorer middle richer richest no_edu primary_ed secondary_ed higher_ed no_radio_listen at_least_once_week_radio_listen always_radio_listen muslim christian other_religion [pw=wgt_indiv] , by(region)

* Save the Weighted Dataset(collpased by region)

save "weighted_merged_data_aug18.dta", replace

* Export Data into csv Format to read into R

export delimited using "weighted_merged_data_aug18.csv", replace

Section 5. Describe the area-level covariates used in statistical analysis. Code written in R version 3.2

#DESCRIPTIVES

data<-read.csv("merged_cov_rates_updated_time_series_revision_august_final.csv")

data$N<-as.numeric(as.character(data$N))

data$D<-as.numeric(as.character(data$D))

data$N[40:42]<-"358335"

data$D[19:21]<-"9811"

library(ggplot2)

library(reshape2)

library(gridExtra)

data$country2 <- ifelse(data$country == "SIERRA LEONE", "SIERRA\nLEONE",

ifelse(data$country == "LIBERIA", "LIBERIA", "GUINEA"))

table(data$country2)

data.m <- data

myplot1<-ggplot(data.m, aes(x = country2, y =rexp , fill =country2 )) + labs(title = "Exponential\nEpidemic Growth Rate")+

geom_boxplot(size=0.5, outlier.size = 0.5) +theme(axis.title.y = element_blank(), axis.title.x = element_blank(), legend.position="none")

myplot2<-ggplot(data.m, aes(x = country2, y =mpoly , fill =country2 )) + labs(title = "Polynomial\nEpidemic Growth Rate")+

geom_boxplot(size=0.5, outlier.size = 0.5) +theme(axis.title.y = element_blank(), axis.title.x = element_blank(), legend.position="none")

myplot3<-ggplot(data.m, aes(x = country2, y =rlogi , fill =country2 )) + labs(title = "Logistic\nEpidemic Growth Rate")+

geom_boxplot(size=0.5, outlier.size = 0.5) +theme(axis.title.y = element_blank(), axis.title.x = element_blank(), legend.position="none")

myplot4<-ggplot(data.m, aes(x = country2, y =female , fill =country2 )) + labs(title = "% Female")+

geom_boxplot(size=0.5, outlier.size = 0.5) +theme(axis.title.y = element_blank(), axis.title.x = element_blank(), legend.position="none")

myplot5<-ggplot(data.m, aes(x = country2, y =age , fill =country2 )) + labs(title = "Mean Age")+

geom_boxplot(size=0.5, outlier.size = 0.5) +theme(axis.title.y = element_blank(), axis.title.x = element_blank(), legend.position="none")

myplot6<-ggplot(data.m, aes(x = country2, y =urban , fill =country2 )) + labs(title = "% Urban")+

geom_boxplot(size=0.5, outlier.size = 0.5) +theme(axis.title.y = element_blank(), axis.title.x = element_blank(), legend.position="none")

myplot7<-ggplot(data.m, aes(x = country2, y =christian , fill =country2 )) + labs(title = "% Christian")+

geom_boxplot(size=0.5, outlier.size = 0.5) +theme(axis.title.y = element_blank(), axis.title.x = element_blank(), legend.position="none")

myplot8<-ggplot(data.m, aes(x = country2, y =muslim , fill =country2 )) + labs(title = "% Muslim")+

geom_boxplot(size=0.5, outlier.size = 0.5) +theme(axis.title.y = element_blank(), axis.title.x = element_blank(), legend.position="none")

myplot9<-ggplot(data.m, aes(x = country2, y =other_religion , fill =country2 )) + labs(title = "% Other Religion")+

geom_boxplot(size=0.5, outlier.size = 0.5) +theme(axis.title.y = element_blank(), axis.title.x = element_blank(), legend.position="none")

myplot10<-ggplot(data.m, aes(x = country2, y =mean_edu , fill =country2 )) + labs(title = "Mean Years of Education")+

geom_boxplot(size=0.5, outlier.size = 0.5) +theme(axis.title.y = element_blank(), axis.title.x = element_blank(), legend.position="none")

myplot11<-ggplot(data.m, aes(x = country2, y =iwi , fill =country2 )) + labs(title = "Wealth Index (IWI)")+

geom_boxplot(size=0.5, outlier.size = 0.5) +theme(axis.title.y = element_blank(), axis.title.x = element_blank(), legend.position="none")

myplot12<-ggplot(data.m, aes(x = country2, y =weekstart , fill =country2 )) + labs(title = "Week from Start EVD")+

geom_boxplot(size=0.5, outlier.size = 0.5) + theme(axis.title.y = element_blank(), axis.title.x = element_blank(), legend.position="none")

myplot13<-ggplot(data.m, aes(x = country2, y =as.numeric(as.character(N))/1000000 , fill =country2 )) + labs(title = "Population Size\n(Millions)")+

geom_boxplot(size=0.5, outlier.size = 0.5) +theme(axis.title.y = element_blank(), axis.title.x = element_blank(), legend.position="none")

myplot14<-ggplot(data.m, aes(x = country2, y =log(as.numeric(as.character(D))) , fill =country2 )) +

labs(title = "Population Density\n(per km sq)") +

geom_boxplot(size=0.5, outlier.size = 0.5) +theme(axis.title.y = element_blank(), axis.title.x = element_blank(), legend.position="none")

myplot15<-ggplot(data.m, aes(x = country2, y =as.numeric(as.character(Numeric)) , fill =country2 )) + labs(title = "Epidemic\nSize") +

geom_boxplot(size=0.5, outlier.size = 0.5) +theme(axis.title.y = element_blank(), axis.title.x = element_blank(), legend.position="none")

# par(mfrow=c(1,3))

# boxplot(as.numeric(as.character(N[which(country=="Guinea")])),col="coral",xlab="Guinea")

# boxplot(as.numeric(as.character(N[which(country=="Liberia")])),col="green",xlab="Liberia")

# boxplot(as.numeric(as.character(N[which(country=="Sierra Leone")])),col="lightblue",xlab="Sierra Leone")

# par(mfrow=c(1,3))

# boxplot(as.numeric(as.character(D[which(country=="Guinea")])),col="coral",xlab="Guinea")

# boxplot(as.numeric(as.character(D[which(country=="Liberia")])),col="green",xlab="Liberia")

# boxplot(as.numeric(as.character(D[which(country=="Sierra Leone")])),col="lightblue",xlab="Sierra Leone")

theme_set(theme_gray(base_size = 15))

tiff("Fig_3_v5.tiff", width = 9, height = 15, units = 'in', res = 300)

grid.arrange(myplot1,myplot2,myplot3, myplot4, myplot5,

myplot6, myplot7, myplot8, myplot9, myplot10,

myplot11,myplot12,myplot13,myplot14,myplot15,

ncol=3,nrow=5)

dev.off()

Section 6. Conduct the area-level statistical analysis. Code written in R version 3.2

data<-read.csv("merged_cov_rates_updated_time_series_revision_august_final.csv")

data$N<-as.numeric(as.character(data$N))

data$D<-as.numeric(as.character(data$D))

data$N[41:42]<-276863

data$N[40]<-338427.228

data$N[27:28]<-358335

data$D[13:14]<-9811

d<-data.frame(

iwi=data$iwi,

edu=data$mean_edu,

christian=data$christian,

muslim=data$muslim,

urban=data$urban,

weekstart=data$weekstart,

N=data$N,

D=data$D,

age=data$age,

female=data$female,

epidemic_size=as.numeric(as.character(data$Numeric)),

rexp=data$rexp,

rlogi=data$rlogi,

mpoly=data$mpoly,

prop_size=as.numeric(as.character(data$Numeric))/data$N)

d1<-d[which(data$uptoweek==6),]

#d2<-na.omit(d[which(data$uptoweek==10),])

d3<-na.omit(d[which(data$uptoweek==15),])

#d4<-na.omit(d[which(data$uptoweek==1000),])

M1<-cor(d1, method = "spearman")

#M2<-cor(d2, method = "spearman")

M3<-cor(d3, method = "spearman")

#M4<-cor(d4, method = "spearman")

cor.mtest <- function(mat, conf.level = 0.95){

mat <- as.matrix(mat)

n <- ncol(mat)

p.mat <- lowCI.mat <- uppCI.mat <- matrix(NA, n, n)

diag(p.mat) <- 0

diag(lowCI.mat) <- diag(uppCI.mat) <- 1

for(i in 1:(n-1)){

for(j in (i+1):n){

tmp <- cor.test(mat[,i], mat[,j], conf.level = conf.level, method = "spearman", exact=FALSE)

p.mat[i,j] <- p.mat[j,i] <- tmp$p.value

# lowCI.mat[i,j] <- lowCI.mat[j,i] <- tmp$conf.int[1]

# uppCI.mat[i,j] <- uppCI.mat[j,i] <- tmp$conf.int[2]

}

}

return(list(p.mat, lowCI.mat, uppCI.mat))

}

library(RColorBrewer)

library(corrplot)

var_names <- c('Wealth', 'Education', '% Christian', '% Muslim', '% Urban',

'Start Week', 'PopSize', 'PopDensity', 'Age', '%Female',

'Epid Size', 'Exp Growth', 'Log Growth', 'Poly Growth', 'Prop Size')

rownames(M1) <- var_names

colnames(M1) <- var_names

tiff("Fig_4_v5.tiff", width = 9, height = 6, units = 'in', res = 350)

par(mfrow=c(1,2))

res1 <- cor.mtest(d1,0.95)

corrplot(M1,type="upper",method="ellipse",col=brewer.pal(n=10, name="RdBu"),xlab="Correlation plot up to week 6", diag=FALSE, tl.col="black",p.mat = res1[[1]], insig = "blank")

# abline(v=10.5)

corrplot(M1,type="upper",method="ellipse",col=brewer.pal(n=10, name="RdBu"),xlab="Correlation plot up to week 6", diag=FALSE, tl.col="black",addrect=2)

# abline(v=10.5)

dev.off()

rownames(M3) <- var_names

colnames(M3) <- var_names

tiff("Fig_S6.tiff", width = 9, height = 6, units = 'in', res = 350)

par(mfrow=c(1,2))

res1 <- cor.mtest(d1,0.95)

corrplot(M3,type="upper",method="ellipse",col=brewer.pal(n=10, name="RdBu"),xlab="Correlation plot up to week 15", diag=FALSE, tl.col="black",p.mat = res1[[1]], insig = "blank")

# abline(v=10.5)

corrplot(M3,type="upper",method="ellipse",col=brewer.pal(n=10, name="RdBu"),xlab="Correlation plot up to week 15", diag=FALSE, tl.col="black",addrect=2)

# abline(v=10.5)

dev.off()

panel.cor <- function(x, y, digits = 2, cex.cor, ...)

{

usr <- par("usr"); on.exit(par(usr))

par(usr = c(0, 1, 0, 1))

# correlation coefficient

r <- cor(x, y)

txt <- format(c(r, 0.123456789), digits = digits)[1]

txt <- paste("r= ", txt, sep = "")

text(0.5, 0.6, txt)

# p-value calculation

p <- cor.test(x, y)$p.value

txt2 <- format(c(p, 0.123456789), digits = digits)[1]

txt2 <- paste("p= ", txt2, sep = "")

if(p<0.01) txt2 <- paste("p= ", "<0.01", sep = "")

text(0.5, 0.4, txt2)

}

#step-wise regression

d<-data.frame(

iwi=data$iwi,

edu=data$mean_edu,

christian=data$christian*100,

muslim=data$muslim*100,

urban=data$urban*100,

weekstart=data$weekstart,

N=data$N/1000,

D=data$D,

age=data$age,

female=data$female*100,

epidemic_size=as.numeric(as.character(data$Numeric)),

rexp=data$rexp,

rlogi=data$rlogi,

mpoly=data$mpoly,

prop_size=as.numeric(as.character(data$Numeric))/data$N,country=data$country)

d1<-d[which(data$uptoweek==6),]

n<-nrow(d1)

fit_exp<-lm(rexp~iwi+edu+christian+urban+weekstart+N+D+age+female+country,data=d1)

step(fit_exp,k=log(n))

fit_exp_sel<-lm(rexp~edu,data=d1)

fit_exp_sel_supp<-lm(rexp~edu+country,data=d1)

fit_logi<-lm(rlogi~iwi+edu+christian+urban+weekstart+N+D+age+female+country,data=d1)

step(fit_logi,k=log(n))

fit_logi_sel<-lm(rlogi~1,data=d1)

fit_logi_sel_supp<-lm(rlogi~country,data=d1)

fit_poly<-lm(mpoly~iwi+edu+christian+urban+weekstart+N+D+age+female+country,data=d1)

step(fit_poly,k=log(n))

fit_poly_sel<-lm(mpoly~edu+urban+N+female,data=d1)

fit_poly_sel_supp<-lm(mpoly~edu+urban+N+female+country,data=d1)

fit_size<-lm(epidemic_size ~iwi+edu+christian+urban+weekstart+N+D+age+female+country,data=d1)

step(fit_size,k=log(n))

fit_size_sel<-lm(epidemic_size ~iwi+edu+N+D+female,data=d1)

fit_size_sel_supp<-lm(epidemic_size ~iwi+edu+N+D+female+country,data=d1)

fit_propsize<-lm(prop_size ~iwi+edu+christian+urban+weekstart+D+age+female+country,data=d1)

step(fit_propsize,k=log(n))

fit_propsize_sel<-lm(prop_size*100 ~edu+female,data=d1)

fit_propsize_sel_supp<-lm(prop_size*100 ~edu+female+country,data=d1)

library(stargazer)

stargazer(fit_exp_sel_supp,fit_logi_sel_supp,fit_poly_sel_supp,fit_size_sel_supp,fit_propsize_sel_supp, ci=TRUE, digits=2, digits.extra=1)

stargazer(fit_exp_sel,fit_logi_sel,fit_poly_sel,fit_size_sel,fit_propsize_sel, ci=TRUE, digits=2, digits.extra=1)
